# Learning New Sensorimotor Contingencies: Effects of Long-Term Use of Sensory Augmentation on the Brain and Conscious Perception

**DOI:** 10.1371/journal.pone.0166647

**Published:** 2016-12-13

**Authors:** Sabine U. König, Frank Schumann, Johannes Keyser, Caspar Goeke, Carina Krause, Susan Wache, Aleksey Lytochkin, Manuel Ebert, Vincent Brunsch, Basil Wahn, Kai Kaspar, Saskia K. Nagel, Tobias Meilinger, Heinrich Bülthoff, Thomas Wolbers, Christian Büchel, Peter König

**Affiliations:** 1 Institute of Cognitive Science, University of Osnabrück, Osnabrück, Germany; 2 Laboratoire Psychologie de la Perception, Université Paris Descartes, Paris, France; 3 Department of Psychology, University of Cologne, Cologne, Germany; 4 Max Planck Institute for Biological Cybernetics, Tübingen, Germany; 5 Aging & Cognition Research Group, German Center for Neurodegenerative Diseases (DZNE), Magdeburg, Germany; 6 NeuroImage Nord, Department of Systems Neuroscience, Hamburg University Hospital Eppendorf, Hamburg, Germany; 7 Department of Neurophysiology and Pathophysiology, University Medical Center Hamburg-Eppendorf, Hamburg, Germany; University of Montreal, CANADA

## Abstract

Theories of embodied cognition propose that perception is shaped by sensory stimuli and by the actions of the organism. Following sensorimotor contingency theory, the mastery of lawful relations between own behavior and resulting changes in sensory signals, called sensorimotor contingencies, is constitutive of conscious perception. Sensorimotor contingency theory predicts that, after training, knowledge relating to new sensorimotor contingencies develops, leading to changes in the activation of sensorimotor systems, and concomitant changes in perception. In the present study, we spell out this hypothesis in detail and investigate whether it is possible to learn new sensorimotor contingencies by sensory augmentation. Specifically, we designed an fMRI compatible sensory augmentation device, the *feelSpace* belt, which gives orientation information about the direction of magnetic north via vibrotactile stimulation on the waist of participants. In a longitudinal study, participants trained with this belt for seven weeks in natural environment. Our EEG results indicate that training with the belt leads to changes in sleep architecture early in the training phase, compatible with the consolidation of procedural learning as well as increased sensorimotor processing and motor programming. The fMRI results suggest that training entails activity in sensory as well as higher motor centers and brain areas known to be involved in navigation. These neural changes are accompanied with changes in how space and the belt signal are perceived, as well as with increased trust in navigational ability. Thus, our data on physiological processes and subjective experiences are compatible with the hypothesis that new sensorimotor contingencies can be acquired using sensory augmentation.

## Introduction

In recent years, theories of cognition underwent profound development in cognitive science [1]. Classical views propose that cognition is precipitated by an internal representation of the outer world shaped by experience [2]. This theoretical framework of cognition, however, fails to satisfactorily explain many aspects of cognition [3]. As a consequence, the developing paradigm of embodied cognition attempts to provide an appropriate and productive framework.

The paradigm of embodied cognition defines cognition as embodied action [4–7]. Even though the approach of embodied cognition involves divers notions [3–5,8], here cognition is understood as an activity that includes mind, body, and environment [3]. Specifically, cognitive processes are conceived as being rooted in the body’s interactions with the world involving perception and action [5]. Embodied cognition in general is theorized as an active and multisensory probing of the environment [9].

Within the framework of embodied cognition, O’Regan and Noë [10] formulated the sensorimotor theory of conscious perception. Sensorimotor theory suggests that learning and mastery of systematic relations of action and associated sensory information, called sensorimotor contingencies (SMC), are constitutive of conscious perception. These “rules or regularities relating sensory inputs to movement, changes and actions” [11] have to be actively learned. The systematic relations between motor action and associated changes of sensory input of modality and object-related SMCs are learned by acting in the world, and concomitantly shape how we perceive the world.

The theory of SMCs is supported by experimental work in the field of sensory substitution. Sensory substitution strives to provide missing or lost sensory information of a specific modality by another substituting modality. In recent years, many studies provided evidence that the brain is able to functionally and structurally adapt throughout life to altered afferent input, to novel experiences due to environmental changes, and to the learning of new skills [12–15]. Already in 1969, Bach-Y-Rita and his colleagues [16] reported that the adult human brain is plastic enough for blind participants to learn how to use a tactile sensory substitution system to perceive visual input and thus recognize and localize objects in the environment. Since then, these results were supported by numerous experiments using vision-to-tactile substitution [17], or other sensory substitution devices like vision-to-auditory substitution [18,19], and vestibular-to-tactile substitution [20]. Learning how to use a sensory substitution device needs time. While subjects learn some perceptual aspects of the substituted stimulus in a very short time, prolonged training with the device develops a more detailed perception [21–23]. However, it has been argued that no true substitution is achieved, and that acquired skills are better described by the analogy to reading [24]. Thus, aspects of substituted as well as substituting modality continue to be relevant. Furthermore, this learning of a new percept much depends on subjects’ active exploration and manipulation with the sensory substitution device improving the richness of the perception with increased quality of the sensation and the action [25]. In line with the SMC theory, the reported perception that was mediated by sensory substitution grew in detail with prolong training duration and active handling of the device.

With the rapid progress of molecular biology even augmenting sensory perception is no longer a theoretical question. It is possible to equip mice with the tool set for color vision [26] or rats with a magnetic sense [27], thus providing them with the means for a new sense. Given this background, the question arises whether humans can learn to perceive sensory information that is not natural in humans. We previously explored this hypothesis using a specially designed sensory augmentation device, called the *feelSpace* belt [28–31]. This device mediates information of magnetic north via continuous vibrotactile stimulation around the waist. Thus, it provides directional information for which humans do not have a natural sensory modality. Sensing the magnetic field is common in the animal kingdom [32–34], but has not been reliably observed in humans. Kaspar et al. [30] showed by evaluating subjective experiences that training with the belt did not lead to a perception of the magnetic field but instead to highly differentiated changes in perception of space of the participants. These perceptual changes included the specific perception of spatial relations of self and objects, an alignment towards cardinal directions that developed to a new feature of objects, and in many participants, to an enlargement of a mental map. Eight out of nine belt wearing participants reported the development of a new sense of spatial perception, which was not found in control participants [30]. Furthermore, the belt’s information could be used in a meaningful way in addition to sensory information that is normally used for navigation, such as visual and vestibular information [35–37]. Even though perceptual and behavioral changes elicited by sensory augmentation are compatible with the SMC theory, four central aspects are still unresolved.

Therefore, in the present study, we used the *feelSpace* belt to develop a deeper understanding of sensory augmentation and test the theory of SMCs. First, to develop SMCs new sensory input provided by an augmentation device has to be actively learned. Therefore, we aimed to obtain insight into the learning process that is involved in using the *feelSpace* belt. As SMC theory predicts that learning and mastering a novel sensorimotor contingency is not dependent on cognitive deliberation, we hypothesize that it involves a procedural learning process. During procedural learning sleep has a beneficial role in processing the information obtained during wakefulness and subsequent memory consolidation [38]. In particular, both in humans [39,40] and in animals [41,42] rapid eye movement (REM) sleep is the most beneficial type of sleep for consolidation of procedural memory [40,42]. Furthermore, intense periods of procedural learning lead to an increase of power in the sigma frequency range during stage 2 sleep and slow wave sleep (SWS) [43]. Accordingly, we hypothesize that training with the *feelSpace belt* will induce a procedural learning process that is reflected by an increase of REM sleep duration as well as by an increased EEG sigma power during sleep, especially in the early training period.

Second, SMC theory predicts that sensory processing cannot be studied in isolation, but by necessity involves motor structures. Thus we hypothesize that learning of the new sensory signal with the *feelSpace* belt induces observable and specific changes in neuronal activity in sensory as well as motor cortex. A number of studies demonstrate neuronal plasticity in healthy subjects through extensive training. For example, right-handed violinists displayed a spatially more extended cortical region relating to the intensively trained left hand while practicing the violin [44]. Similarly, the investigation of London taxi drivers revealed a larger hippocampus associated with their huge amount of navigational abilities and map knowledge [45]. Several studies examining learning and use of sensory substitution devices [13,46,47] in sighted and blind humans observed physiological changes in brain activation patterns with cross-modal activation and activation of higher cortical areas. Along similar lines, we compare the brain activity before and after the training with the *feelSpace* belt in a fMRI paradigm. As the concept of mastering SMCs proposes a direct relevance of motor action for processing of sensory information, we hypothesize that learning to utilize the *feelSpace* belt as a sensory augmentation device involves not only low-level somatosensory areas but also higher order motor centers and areas that are involved in navigation. Specifically, acquiring and mastering the belt’s information will induce changes in brain activity involving higher motor centers, such as supplementary motor area and posterior parietal cortex, and brain regions participating in navigation, like the nucleus caudatus, and the hippocampus.

Third, we hypothesized that once the new sensory input is mastered; it can be actively used and is observable in behavioral tasks. Path integration, firstly postulated by Charles Darwin [48], enables homing back to the starting position during the exploration of a new environment or in commuting between a nest and familiar feeding grounds e.g. [49,50]. Species who rely on path integration for foraging typically use global heading information obtained from polarized light or the Earth’s magnetic field [51–53], which reduces error accumulation during path integration [54]. As suggested by Nagel et al. [28], humans are also able to use the information of the magnetic north provided by the *feelSpace* belt in a triangle completion task. Expanding the concept of that task, we designed a complex homing task to provide more natural experimental conditions for testing behavioral changes with the *feelSpace* belt. We therefore hypothesize that humans who are provided with the information of the magnetic north via the *feelSpace* belt will be able to use this information in a complex homing task.

Fourth, compatible with the sensorimotor contingency theory that postulates that mastery of new SMCs will be accompanied by changes in the perception of the world [10,55,56], we hypothesize to find perceptual changes after the training period with the belt. Using a mixed-method approach of subjective evaluations e.g., [57] we assessed changes in subjective experiences and quantified the extent of changes over the training duration with the *feelSpace* belt. As the belt provides additional navigational information through translating the information of the magnetic north into a tactile signal, we examined both the perception of space in which participants navigate and the perception of the belt’s signal itself. We predict that in the course of training with a sensory augmentation device perception of space as well as of the tactile signal will increasingly be modified.

Consequently, we present a longitudinal investigation involving a seven-week training period with a sensory augmentation device in natural environment as a test several predictions of the SMC theory. In four experiments we explore the hypotheses (H) that learning and mastering the new augmentation signal: (H 1) induces a procedural learning process, (H 2) induces changes in brain activation patterns including the activation of higher motor areas and areas related to navigation, (H 3) will be observable in behavioral changes in a complex homing task, and (H 4) will lead to perceptual changes of space and the tactile belt signal, which are correlated with the training duration. Although, using the sensory augmentation device behavioral results did not reach significance, both EEG and fMRI measurements provided results compatible with the SMC theory and subjects reported significant perceptual changes.

## Results

### Participants

Nine participants (19–32 y, *mean* 23.67 y, four females) wearing the *feelSpace* belt all waking hours formed our experimental belt wearing group and five additional participants (21–25 y, *mean* 23.00 y, three females) not wearing a belt formed the control group.

All participants were healthy young adults, without neurologic, psychiatric, or chronic diseases. They were highly motivated and had good introspection and good verbal skills (for details see [30]). They were selected such that prior to the study they performed plenty of outdoor exercises such as hiking and bicycling. Both groups were asked to continue outdoor activities dedicating them to an unsupervised navigational training during the seven week training period for at least 1.5 h/d in natural environment. Belt wearing participants were asked to explore the belt signal during their navigation activities, while control participants were asked to observe how they navigated during their navigation activities. Both groups were asked to pay attention to their space perception while moving in natural environment.

To ensure that dedicated outdoor navigation (from now on called “training”) and motivation were similar in both groups, belt wearing and control participants recorded their daily training duration and scored their weekly training motivation. The training duration with the belt averaged across the training period of 7 weeks was 1.57 h/d (*SD* = 0.17). The control group performed the navigation training without the belt for the same amount of time (1.57 h/d, *SD* = 0.55). Both groups rated their weekly training motivation with and without a belt, respectively, over the whole training duration as very high (grand mean of 3.97 on a 5 point Likert scale; *SD* belt = 0.30, *SD* control = 0.34) [30]. Thus, belt wearing and control participants showed a high training motivation with no differences in motivation and training duration between groups.

All participants were extensively briefed in a dedicated meeting and provided informed, written consent before participating.

### The *feelSpace* belt

**Fig 1:**
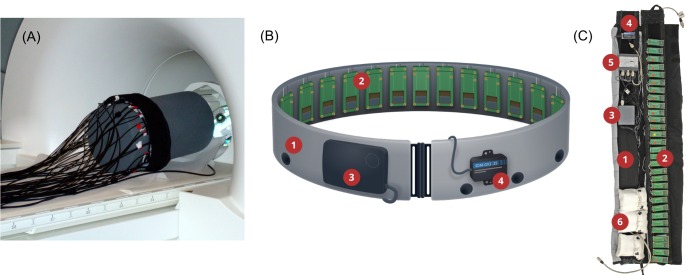
The sensory augmentation device. (A) For testing and demonstration purposes the MRI *feelSpace* belt in the scanner is wrapped around a dummy with cables connecting the vibrating piezo actuators to an external computer. (B, C) The portable *feelSpace* belt (1) consists of the following main components: 30 vibrotactile piezo actuators that are identical to those of the MRI compatible belt (2), compass-control unit (3), an electronic compass (4), piezo-control unit with identical control as the scanner unit (5), and battery packs (6). Figure A taken from Keyser [58] and under Creative Commons CC-BY-3 from Schumann [31].

The augmentation device, which we used in this study, is a further development of the *feelSpace* belt designed by Nagel et al. [28]. This belt gives directional information about magnetic north via vibrotactile stimulation around the waist. Here, we developed a special MRI-compatible version of the *feelSpace* belt using non-ferromagnetic piezo-ceramic actuators to study the neural correlates of SMC learning (Fig 1A). An accompanying set of novel portable *feelSpace* belts uses the identical piezo-ceramic actuators and ensures that vibrotactile stimulation is identical in the scanner and in everyday training with the belts (Fig 1B and 1C). Portable belts in addition contain a control unit, an electronic compass and battery packs to function independently and to allow free movement while wearing the belt (see method section for more details).

### Study design

Before the start of the training period, we conducted baseline measurements for all participants recording sleep EEG, fMRI, and a behavioral homing task and performed specifically designed and standardized questionnaires as well as interviews [30]. At this time point both groups revealed comparable results (see in detail in the separate result sections). The sequence of these measurements was chosen according to the availability of the fMRI and sleep EEG labs. The same measurements were performed at the end of the seven-week training period (see Table 1). We chose seven weeks for our training period as a trade of between reasonable burden for the participants and a time period sufficient for the induction of observable effects [28–31]. In addition to the experiments proper and to the self-guided everyday navigation training, all participants took part in a supervised weekly outdoor training. The outdoor trainings had a large scope from angle turning training to a “treasure hunt” in a natural environment. The study was performed in four cohorts each lasting for eight to nine weeks with a seven-week training period.

**Table 1:** Timetable of measurements.

Before Training	Main Training Period	Last Week of Training
Sleep EEG	Sleep EEG (night 1 and 4)	Sleep EEG
fMRI		fMRI
Homing		Homing
Questionnaires	Daily and weekly Questionnaires	Questionnaires
Interview	Weekly Interview	Final Interview

Our study complied with Helsinki Declaration guidelines and was approved by the ethics committee of the University Osnabrück.

### Hypothesis 1: Training with the *feelSpace* belt involves procedural learning, observable in neuronal signatures of sleep

According to previous studies, learning-dependent changes are reflected in general sleep architecture and tonic EEG activity [41–43]. Specifically, procedural learning induces an increase of REM sleep duration and changes of power in the sigma frequency range during stage 2 sleep and slow-wave sleep. Thus, we performed sleep EEG measurements and recorded four nights per participant. Belt-induced learning was expected to be most prominent in the early training phase. Therefore, we recorded a baseline night before the start of the study, two additional nights at the beginning of the training period (first and fourth night), and one night in the last week of the training period. We excluded one participant from the analyses due to poor sleep quality (i.e. more than 20% of awake and 20% of stage 1 sleep in the EEG data throughout the baseline measurement and the first test night). Four independent coders rated the sleep EEG data following the standard sleep scoring criteria with an inter-rater reliability of above 90% [59].

**Fig 2:**
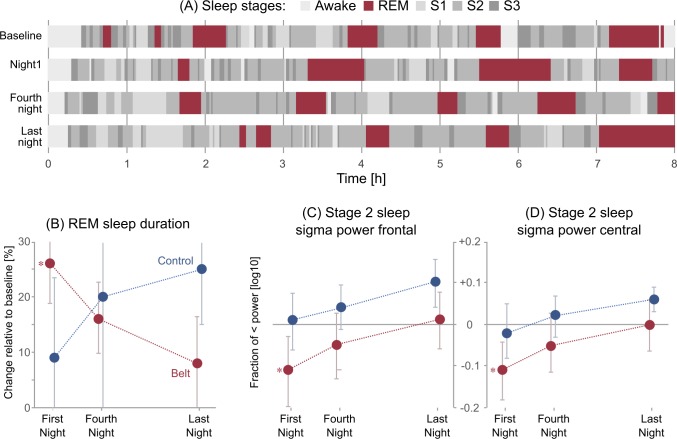
Single participant and group statistics of sleep EEG. (A) Hypnograms of a belt wearing participant demonstrating the distribution of sleep stages during the nights (abscissa) before and during training (top to bottom). REM sleep phases are marked in red. (B) Relative changes in the REM sleep duration for all belt wearing participants and controls during the training period. (C, D) Learning-dependent changes in the sigma power (12–16 Hz) during Stage 2 sleep at frontal (C) and central (D) electrodes in belt participants and in controls. Error bars depict SEM. Please note that SEM is influenced by variance as well as group size. An asterix indicates a significant effect.

The sleep parameters showed a skewed distribution, and we used a one-tailed permutation test with 10^5^ samples for the analysis. The baseline measurement showed no significant differences in sleep stage durations between the belt wearing and the control group (belt vs. control REM sleep duration difference *p* = 0.46, for all other sleep stage durations *p* values range from 0.29 to 0.46). Further results revealed changes in sleep architecture in the belt wearing group after onset of the belt training (Fig 2A and 2B). In particular, we found a significant increase of REM sleep duration in the belt wearing group (*p* = 0.037) in the first night after training onset compared to the baseline night. This increase of REM sleep returned to the baseline level towards the end of the training period. Additionally, we found a decrease of stage 1 sleep in the first night of the training period compared to the baseline measurement (*p* = 0.037). In contrast to the belt wearing participants, control participants showed an increase of REM sleep duration towards the end of the training duration, which did not reach significance (*p* values ranging from 0.119 (baseline—last night) to 0.412). Furthermore, control participants showed no significant changes in stage 1 sleep (*p* values range from 0.117 to 0.41) and we observed for both groups (for belt group: *p* values range from 0.256 to 0.454 and for control participants: *p* values range from 0.110 to 0.345) no significant changes for Non REM sleep parameters (stage 2).

To further analyze learning-dependent changes relative to the baseline night in the EEG spectrum, we focused our power spectrum analysis on three frequencies (delta: 0.5–4 Hz, theta: 4–8 Hz, sigma: 12–16 Hz) that are representative of SWS, REM, and stage 2 sleep phases, respectively. Comparing to the baseline data we performed a separate spectral EEG analysis computing a 2 x 3 x 2 (group x night vs. baseline x electrode placement) mixed-measures ANOVA. For the electrode placement we concentrated on frontal and central electrode placement [60,61]. For the baseline night we compared the belt wearing group and the control group with an independent t-test, which revealed no significant differences between groups before the training (delta power: *t*(11) = -1.473, *p* = 0.169, theta power: *t*(11) = 0.195, *p* = 0.849, sigma power: *t*(11) = 0.761, *p* = 0.462). For the delta and theta frequency bands the ANOVA revealed no significant main effects for group, night or electrode placement (all F ≤ 3.129, p ≥ 0.105), nor significant interactions (all F ≤ 1.451, p ≥ 0.254). However, in the sigma frequency band we found a significant between-group effect (*F*(1, 11) = 5.358, *p* = 0.041) and a significant within-group effect for nights (*F*(2, 22) = 7.878, *p* = 0.003). A follow-up analysis using paired t-tests revealed a significant decrease in sigma power from the baseline to first night of the belt wearing group (*t*(7) = 5.587, *p* = 0.001), (Fig 2C and 2D). Towards the end of the training, sigma power values returned to their baseline levels reflected in the means (mean baseline = 0.4204, mean last night = 0.4239) and in a significant increase from the first night to the last night (*t*(7) = -3.792, *p* = 0.007). For the control group no significant changes in sigma power over nights were observed (*t*(4) = -0.116, *p* = 0.913, *t*(4) = 0.855, *p* = 0.441 and *t*(4) = 1.289, *p* = 0.267, for first, fourth and last night respectively). Due to the small sample size, we also validated all results by using a non-parametric Friedman-test suitable for small sample size analyses. All results could be replicated.

Summarizing, the sleep EEG measurements revealed in the belt wearing group a significantly increased REM sleep duration indicative of procedural learning and significant EEG sigma power decrease indicative of sensorimotor processing early in the training period.

### Hypothesis 2: Training with the *feelSpace* belt induces changes in cortical activation, in particular in motor centers and brain regions involved in navigation

We investigate the physiological basis of sensory augmentation via fMRI measurements. During the recordings the participants viewed a minimalistic virtual environment from a first-person perspective on a monitor. Participants performed a virtual *homing* task, and a *control task* with identical visual and tactile stimuli (adapted from [62]). All participants were wearing the fMRI compatible version of the belt during the recordings. In half of the trials of either task the belt was switched on coherently indicating participants’ virtual direction towards an arbitrarily but consistently defined virtual north via vibration. In the other half of all trials the belt was switched off to measure path integration abilities in the absence of the belt information. This resulted in a four factor (2 x 2 x 2 x 2) design: *belt* (on/off), *task* (homing/control), *group* (belt wearing/control group), and *date* (before/after training). Here, we concentrate on the four main effects and the significant two-way interactions.

**Fig 3:**
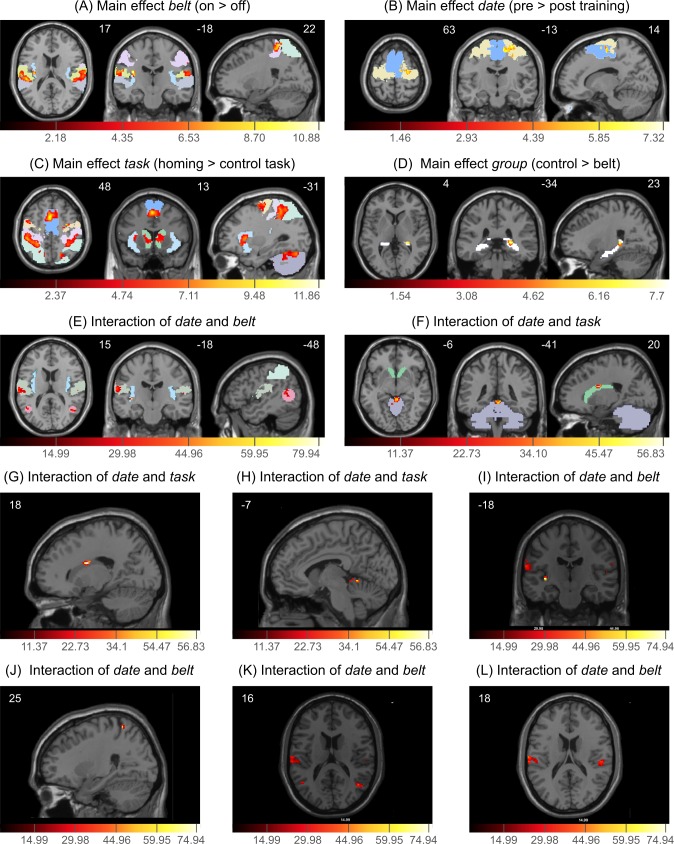
Main effect and 2-way interactions in the fMRI data. Significant BOLD activation differences (the activation color-coding is depicted below the figures) in ROIs in z-, y-, x-planes (coordinates are depicted on top of each panel). (A) Main effect *belt* (on > off), (B) Main effect *date* (pre > post training), (C) Main effect *task* (homing > control task), (D) Main effect *group* (control > belt), (E) Interaction of *date* and *belt*, (F) Interaction of *date* and *task*. Captions color-coding ROIs in Fig 3(A)-(F): S1 lavender, S2 khaki, PPC light green, STG light gray, Insula baby blue, premotor cortex peach, SMA blue, cerebellum dark gray, caudate nucleus forest green, Hippocampus white, MST pink. (G-L) Blown ups of planes best depicting peak activations in ROIS of significant BOLD activation differences in two-way interactions: (G) Interaction *date x task*, peak activation of caudate nucleus, (H) Interaction *date x task*, peak activation of Cerebellum, (I) Interaction *date x belt*, peak activation of insula, (J) Interaction *date x belt*, peak activation of PPC, (K) Interaction *date x belt*, peak activation of MST, (L) Interaction *date x belt*, peak activation of S2, (S2 also depicted in I and K).

For the analysis we used a mixed-effects ANOVA to decompose the four factors and report the main effects of BOLD activation and two-way interactions. We defined regions of interest (ROIs) to concentrate on areas relevant for sensorimotor processing and spatial navigation including higher-level regions. We chose the ROIs considering areas previously defined in similar studies (e.g. [62–65]). The ROIs were analyzed, and results are reported for clusters larger than 5 voxels when the activation difference was significant with *p* < 0.05 (FDR corrected). Table 2 gives an overview of ROIs and significant activations. Fig 3 shows the canonical MNI T1 weighted anatomical image as a backdrop. Superimposed on the structural scans are the activation differences in the ROIs with *p* < 0.001 (uncorrected). Coordinates are given in MNI space and definitions and labeling of the ROIs were mainly taken from the Anatomical Automatic Labeling atlas from the Wake Forest University Pickatlas [66] and the anatomy toolbox of SPM [67]. In the figures, we show the planes where the highest number of ROIs and significant activation differences are visible. Thereby, peak activation differences of single ROIs may lie in adjacent planes. All figures show the three planes in z, y, x-coordinates of MNI-space.

**Table 2:** Table of defined ROIS and overview of activations.

	Factors					
ROIs	Belt	Task	Date	Group	Date*belt	Date*task
**S1**	x	x	--	--	--	--
**S2**	x	--	--	--	x	--
**PPC**	x(r)	x	--	--	x	--
**MST**	--	x	--	--	x	--
**STG**	x	--	--	--	--	--
**Insula**	x	x	--	--	x	--
**Hippocampus**	--	--	--	x	--	--
**Premotor cortex**	--	x	x	--	--	--
**SMA**	--	x	x	--	--	--
**Primary motor cortex**	--	--	--	--	--	--
**Cerebellum**	--	x	--	--	--	x
**Caudate**	--	x	--	--	--	x

(x = significant activation with p < = 0.05 (FDR corrected), - = no significant activation, r = right)

The main effect *belt* (Fig 3A) compares brain activation between the condition where the tactile belt is switched on versus the condition where the belt is switched off. Investigating the main effect *belt*, we observe in the *on* condition a significantly higher bilateral activation as in the off condition in the primary somatosensory cortex (S1) and secondary somatosensory cortex (S2) as well as the insula, superior temporal gyrus (STG), and the right posterior parietal cortex (PPC). Thus, we see significant activation of somatosensory regions when the tactile signal of the belt is given.

The main effect *date* (Fig 3B) contrasts the activation difference between the pre- and post-training date. The main effect *date* shows a differential activation of the premotor cortex and the supplementary motor area (SMA). Specifically, after the training period the BOLD signal in motor areas is reduced. The effect date reveals an activation difference between before and after training in higher order motor areas.

The main effect *task* (Fig 3C) compares the brain activation differences between the virtual homing task and the control task. In this main effect we found a significant activation difference in a large sensorimotor network, including S1, the PPC, medial superior temporal cortices (MST), the insula, the premotor area, SMA, and the cerebellum. Additionally, the differential activation uncovers a higher activation for the homing task in the caudate nucleus. We found differential activation of a large sensorimotor network and of areas that are known to be involved in navigational aspects.

The main effect *group* (Fig 3D) compares the activation differences between the belt wearing and the control group. On average, the control participants show higher activation of the hippocampus than the belt wearing participants. The main effect of *group* shows a difference in right hippocampus activation between the two groups.

The evaluation of the two-way interactions revealed significant activation differences in the interactions of date*belt and date*task. The remaining interactions (group*date, group*belt, group*task and belt*task) did not show significant activation differences in the predefined ROIs.

The interaction of date*belt (Fig 3E) compares activation differences of *belt* signal *on* and *off* contingent on the time of recording before or after the training. Significant activation differences in the interaction date*belt were observed in regions S2, PPC, MST, and the insula. Specifically, the activation of S2 revealed significant differences in the *belt on > off* condition only in the first measurement before the training period. After the training period there is no significant activation difference between the *belt on* and *belt off* condition in S2. We found a reduction of belt on/off activation differences after the training especially in the secondary somatosensory cortex.

The interaction date*task (Fig 3F) compares activation differences in the *homing* and *control* task before and after training with the *feelSpace* belt. In this interaction we found significant differential activation in the cerebellum and the caudate nucleus. In the cerebellum, before the training period the *homing* and the *control* task revealed no significant activation differences. However, after the training we observe a significantly higher activation in the *homing* than in the *control* task. In the caudate nucleus we found no activation difference before and after training in the *control* task compared to the homing task, which reveals a higher activation in the caudate nucleus before compared to after training. These data reveal a time dependent activation pattern difference in the cerebellum and caudate nucleus for the homing and the control task.

In summary, the fMRI measurements indicate that sensory augmentation by means of training with the *feelSpace* belt involves differential activation patterns including sensory areas (S1, S2), higher motor centers (premotor cortex, SMA, PPC, STG, Insula), cerebellum, and brain areas known to be involved in navigation (hippocampus, caudate nucleus).

### Hypothesis 3: After training with the *feelSpace* belt participants successfully utilize the belt in a complex homing task

**Fig 4:**
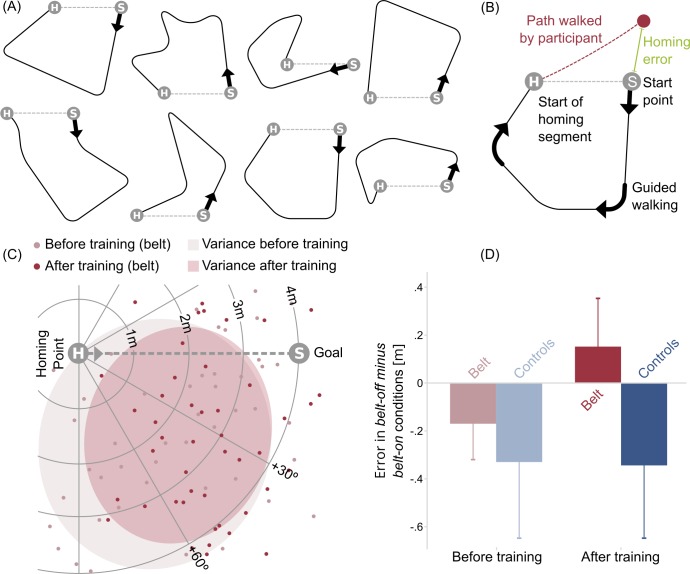
Layout of the homing task. (A) Design of the polygons used in the homing task. (B) Example polygon depicting the homing error (error between start point and actual end point of participants walk), H = Homing point, point from which participants had to home to the starting point on their own, S = Start point, red dot = actual end point of participants’ path, grey dashed line H-S = ideal homing segment. (C) Pre- to post-training comparison of homing errors of belt wearing participants in the *belt*-*on* condition. For visualization polygons were superimposed and mirrored and/or rotated in a way that all of them end up below the dashed line which represents the optimal path from homing point (H) to starting point (S). An inward error could be observed in the position of the ellipse underneath the homing trajectory could be observed. (D) Effect of belt use (error in *belt-off minus belt-on* conditions, positive numbers indicate a reduction of homing error) onto homing error comparing pre- to post-measurement for belt wearing and control group. Error bars indicate SEM.

To investigate behavioral changes with the belt we designed a homing paradigm as an extension of the conventional triangle completion task. It consisted of eight carefully crafted, complex, curvy polygons without intersections (Fig 4A). The design of the figures was supposed to trigger continuous updating (for more detail see method section). While solving the homing task, participants additionally had to memorize numbers as a cognitive load.

Our main variable was the distance between starting point (Fig 4B, S) and the point where the participant finished homing (Fig 4B, red dot)), the so-called homing error. A separate analysis of the angular error led to identical results. For the analysis we rotated polygons and mirror reversed them as necessary to align the optimal homing trajectories (Fig 4A; black dashed line from H to S with the polygon below). To investigate comparability between groups at the beginning of the training period we performed an independent t test for the baseline measurement date before the start of the training. We found no significant difference between the belt wearing and control group (*F* = 2.901, *p* = 0.114) at this time point. To evaluate the effect of the belt information on the navigation performance, we visualized the difference of homing error in the belt-off minus the belt-on condition for both groups before and after the training period (Fig 4D). Our data show a decrease in performance in the pre-measurement when the belt information was given in both groups, hypothetical because of the additional unknown sensory input. This effect of the belt signal is reversed after training only in the belt wearing group.

For statistical evaluation of navigational performance we performed a 2 x 2 x 2 (*date*, *belt*, and *group*) mixed-measures ANOVA. However, the results revealed no significant main effects of *group* (*F*(1,12) = 3.425, *p* = 0.089), *date* (*F*(1,12) = 3.066, *p* = 0.105), or *belt* (*F*(1,12) = 1.446, *p* = 0.252), nor for interactions (*F*(1,12) between 0.298 and 1.217, *p* values between 0.595 and 0.292).

Summarizing, our results showed no significant change in performance neither for belt wearing nor control group.

### Hypothesis 4: Learning of new SMCs with sensory augmentation leads to perceptual changes that are positively correlated with training duration

To assess subjective perception we developed daily and weekly questionnaires with supplementary weekly interviews for in-depth evaluation of participants’ experiences. To obtain qualitative and quantitative estimations of changes questionnaires contained open-ended questions and 5-point Likert items (“not agree” (1), “a little agree” (2), “more or less agree” (3), “quite agree” (4), to “very agree” (5) or “never” (1), “seldom” (2), “sometimes” (3), “often” (4), to “always” (5)). In the questionnaires of the control participants’ questions were phrased in close analogy to the belt questionnaire substituting “with the belt” by “since I train my orientation” in questions not referring to the belt signal experience as such. Apart from this, we kept the phrasing of the questions equal. Special items referring to the belt signal experience had to be excluded in the control questionnaire, naturally (for more details see the method section below).

Additionally, we evaluated the German version of the NEO-FFI [68] and the ACS-90 [69] to asses relevant personality traits and the “Fragebogen Räumliche Strategien” (FRS) [70] to asses navigational behavior. Participant groups did not significantly differ in personality traits (Mann-Whitney-U-test for NEO-FFI: all Z = -1.67 or smaller, all p = 0.11 or bigger and for ACS-90: all Z = -1.17 or smaller, all p = 0.30 or bigger). In the FRS for the baseline measurements groups also did not significantly differ (Mann-Whitney-U-test all Z = -0.67 or smaller, all p = 0.52 or bigger). For the belt wearing group we measured the AttrakDiff2 [71] questionnaire to assess the *feelSpace* belt. These latter questionnaires and the qualitative data of our daily and weekly questionnaires have been thoroughly analyzed and published elsewhere [30].

For the analysis of our quantitative data we performed a factor analysis of the Likert items of the weekly questionnaire to evaluate underlying factors. For the factor analysis, we used the Guttman-Kaiser Criterion for factor extraction (factors with eigenvalues larger than 1 were extracted) and used a varimax rotation to rotate the factor matrix. As an item analysis, we calculated mean, variance, and selectivity as well as the item-total correlation for all items. Based on these analyses, no item had to be excluded for the following factor analysis.

The factor analysis of quantitative items resulted in four factors for the belt wearing group. The factor loadings were evenly distributed across these four factors and they jointly explained a major fraction of the variance (see Table 3). Furthermore, the communalities of the individual items ranged between 0.61 and 0.97, resulting in an average of 0.84. The internal consistencies of items (Cronbach alpha) were between 0.62 and 0.85, with an average of 0.79 for the factors of the belt wearing group and 0.67 for the factor of the control group. The item “Since wearing the belt I am more aware of the cardinal directions.” was removed in the statistic from the factor “*space perception*” to allow comparability between the belt wearing and control groups (see below). We choose the labels for the factors due to the common topics of the respective questions of the questionnaire: *space perception* (#1), *trust in navigational ability* (#2), *tactile belt perception* (#3), and *conscious belt perception* (#4). For the control group the factor analysis added up to one factor only. This factor had the largest overlap with the factor *space perception* of the belt group. Therefore we took the intersection and considered only the joint items in the following. The factor analysis revealed a good mapping of our 13 items onto four factors for the belt wearing group (see Table 3) and onto one factor for the control group where we only considered the 3 corresponding items matching the factor *space perception* of the belt wearing group.

**Table 3:** Factor analysis of quantitative data of the weekly questionnaire for the belt wearing group.

	Loading on Factors				
Questions defining the items	#1	#2	#3	#4	r^2^
With the belt I can give more precise estimations on how streets are related to one another.	0.88	-0.28	0.00	0.07	0.86
With the belt it is easier for me to indicate the position of different places to each other.	0.84	0.33	0.22	0.19	0.90
With the belt I am always aware where I am located in relation to my home.	0.70	-0.43	0.40	-0.11	0.85
Since wearing the belt I am more aware of the cardinal directions.	0.65	0.28	-0.05	-0.41	0.67
I have the feeling that my spatial sense of orientation improved since wearing the belt.	0.26	0.93	-0.03	-0.09	0.95
With the belt I feel safer in a new environment than without the belt.	-0.26	0.87	0.10	0.09	0.85
With the belt it is easier for me to orient myself in a new environment than without the belt.	-0.09	0.74	-0.21	-0.05	0.61
When I take the belt off my spatial sense of orientation decreases.	0.24	0.66	0.10	-0.60	0.85
I do not perceive the transmitted information of the belt as vibration but as something different.	-0.08	-0.15	0.91	-0.27	0.93
I perceive the transmitted information as vibration.	0.25	0.36	0.87	0.16	0.97
After taking the belt off I still perceive a feeling of vibration.	0.17	-0.22	0.83	0.28	0.84
I consciously concentrate on the belt to use its information.	0.03	-0.28	0.03	0.87	0.85
I am always consciously aware of the belt while wearing it.	0.04	0.39	0.09	0.78	0.77
Explained variance [%]	20.76	26.86	19.55	16.58	83.75

The last column (**r2**) gives the communality of the factor loading for each item.

For comparability we give here the corresponding item phrasing of the three items loading on the factor “space perception” for the control group: 1. “Since I train my orientation I can give more precise estimations on how streets are related to one another.” 2. “Since I train my orientation it is easier for me to indicate the position of different places to each other.” 3.” I am always aware where I am located in relation to my home. “. To give an insight into the precise reports by the participants, we give here examples of citations for the main categories of the qualitative content analysis [30] of belt wearing participants (BWP) and control participants (CP).

- The following citations give evidence of a profound change of *space perception*. “*Each place in space has now*, *depending on how I am located to it*, *an additional information*, *which I can’t yet connect globally*” (BWP 1, week 1). “*Space is getting wider and deeper*. *Through the presence of objects/landmarks that are not visible my space perception is extending beyond the borders of what I see*” (BWP3, week 6). “*The direction is one information more that is always available*. *This direction information is no specification of other signals (visual or mental maps)*, *but is really independent thereof*” (BWP 9, week 6).
- The increased *trust in navigational abilities* is indicated for example by a participant who stated, *“Because of the permanent knowledge of the northern direction a new space perception develops*, *a feeling of security”* (BWP 4, week 6).
- The *tactile belt perception* is related to space, “*I perceive* [the vibration of the belt] *as an existent information about the northern cardinal direction*” (BWP 4, week 4). *“I perceive the information of the belt as a pointer towards the north direction or a pointer towards places*, *e*.*g*., *the university or my desk at home”* (BWP 8, week 2).
- The *conscious belt perception* is clearly changing over time. *“I have the feeling that I don’t use the belts information consciously not only in known areas any more*, *but that I use and feel the signal consciously when I need it*. *And I need it (…) when I have to orientate myself”* (BWP 8, week 5). “*Once again I noticed that I just use it* [the belt signal] *without being aware of it”*(BWP 7, week 6).
- In contrast, statements by control participants give evidence that they have to concentrate for navigation, “*My mental map didn’t change a lot*. *But I am more aware of it*”(CP 5, week 4). “*If I concentrate a lot I can integrate other things*, *e*.*g*., *cardinal directions into my space perception*. *But without deliberately doing this nothing changes*” [German original: aber ohne das bewusst zu machen ändert sich nichts.] (CP 2, week 5).

Thus, the statements of the participants given in the interviews or diaries fully support the factor analysis of the quantitative questionnaires.

**Fig 5:**
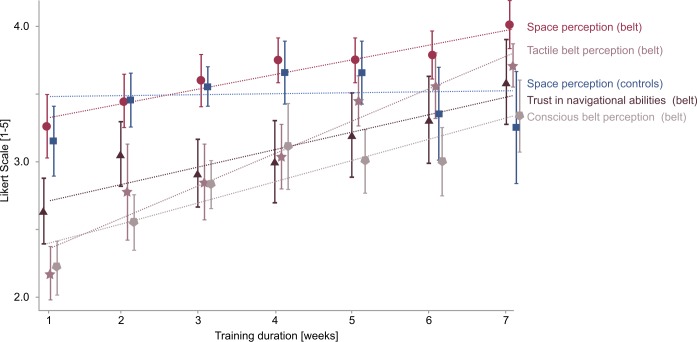
Factor ratings for the belt wearing and control group. Ratings for factors *trust in navigational ability* (black triangles), *tactile belt perception* (pink stars), *conscious belt perception* (light pentagons) and *space perception* (red circles) for the belt wearing group and *space perception* (blue squares) for the control group as a function of weeks. Ratings range from 1 to 5 (not agree to very agree), indicating a low or high rating for the factor, respectively. Dotted lines indicate fitted lines by a linear mixed model for each factor. Error bars are SEM.

Following the factor analysis, we investigated the changes of the ratings for the four factors as a function of the training duration for the belt wearing group (Fig 5). In order to test whether ratings for the factors *conscious belt perception*, *belt information*, *trust in navigational ability* and *space perception* increase over time, we fitted separate linear mixed models for each factor with the continuous predictor weeks. To account for the dependence between measurements within individual participants, we modeled individual intercepts for each participant [72]. We found that all models yielded significant positive slopes, indicating an increase in ratings over time (Fig 5). The highest increase in ratings is found for the factor *tactile belt perception* (*B* = 0.24, *p* < 0.001, 95% CI [0.18, 0.30]), followed by the factors *conscious belt perception* (*B* = 0.16, *p* = 0.001, 95% CI [0.10, 0.21]), *trust in navigational ability* (*B* = 0.13, *p* = 0.017, 95% CI [0.07, 0.18]), and *space perception* (*B* = 0.11, *p* = 0.002, 95% CI [0.07, 0.15]). Using for this factor the same items for the belt wearing group as for the control group did not change the result (*B* = 0.11, *p* = 0.003, 95% CI [0.05, 0.17]). When we performed the same analysis for the factor of *space perception* in the control group, we did not find a significant slope (*B* = 0.007, *p* = 0.90, 95% CI [-0.12, 0.14]), indicating that participants’ *space perception* ratings in the control group did not increase over time. In the belt wearing group, factor ratings for all four factors increased significantly over time whereas in the control group ratings for the (only) factor *space perception* did not change over time.

In addition, we investigated the difference in ratings for the factor of *space perception* between the belt wearing and the control group over the training duration (Fig 5). To investigate comparability between groups at the beginning of the training period we performed an independent t test for the first measurement date after one week of training. We found no significant difference between the belt wearing and control group (*F* = 0.071, *p* = 0.794) at this time point. We then compared whether participants in the belt wearing group showed a steeper increase in *space perception* ratings over weeks in comparison to the control group. For this purpose, we examined how the slopes as a function of weeks differed between the groups using a linear mixed model. We found that the slope is significantly larger for the belt wearing group in comparison to the control group (*B* = 0.10, *p* = 0.005, 95% CI [-0.17, -0.03]). Belt wearing participants’ rating increase for the factor *space perception* over weeks was significantly larger than in the control group.

Taken together, we observed a continuous increase of the ratings of all factors (*space perception*, *trust in navigational ability*, *tactile belt perception*, *and conscious belt perception*) over the training duration in the belt wearing group. The longer the belt wearing participants wore the belt the higher they rated all factors indicating a continuous evolution of changes in subjective experiences over time. In contrast, the rating for the factor (*space perception*) in the control group showed no systematic change over time. The comparison of the factor *space perception* between belt wearing and control groups revealed a significantly higher rating increase in the belt wearing group over weeks. Therefore, we can conclude that the training with the *feelSpace* belt led to increasing changes of subjective experiences of perception of space and perception of the belt signal and trust in navigational ability over time.

## Discussion

In the present study, we experimentally tested several predictions of the SMC theory by observing the learning and mastery of a new sensory signal given with a sensory augmentation device. The increased REM sleep duration in the early training phase with the *feelSpace* belt indicated an intensified procedural learning process (hypothesis 1). The decrease in sigma power only in the belt wearing group at the same time suggested increased sensorimotor processing. FMRI measurements revealed during a virtual homing task a differential activation of sensory and higher motor areas, i.e., PPC, SMA, premotor cortex, and brain areas known to be involved in navigation (hypothesis 2). Furthermore, after seven weeks of training neither the belt wearing nor the control group showed a significant increase in performance during a complex navigation task (hypothesis 3). Evaluation of participants’ subjective experiences, while using the belt, indicated a continuous evolution of changes with increasing training duration in the perception of space and of the belt signal, as well as an increasing trust in navigational abilities when using the belt (hypothesis 4). Evaluation of control participants’ reports revealed no changes of their spatial perception over time. In summary, our results provide no support for hypothesis 3, but give evidence that the present kind of sensory augmentation leads to procedural learning, involvement of motor areas and areas involved in navigation with concomitant perceptual changes in subjective experiences. Our experimental results comply with predictions of learning new sensorimotor contingencies.

### Procedural learning

Our hypothesis that training with the *feelSpace* belt induces procedural learning was supported by sleep-EEG measurements. Here we found a significantly increased fraction of REM sleep duration in participants training with the *feelSpace* belt with a maximum in the first training night whereas control participants showed no significant changes of REM sleep duration over the training period. Previous studies on humans [38,39] and animals [41,42] reported that procedural learning leads to an increased REM sleep duration. Once animals mastered performance of these tasks, REM levels returned to normal [73,74]. This observation is in line with our results, showing that REM sleep duration in belt wearing participants returned to the level of the baseline night by the end of the seven weeks training period. Taken together, the changes of REM sleep duration suggest that training with the *feelSpace* belt induces a procedural learning process.

To further investigate the procedural learning process we also evaluated tonic EEG activity during sleep. Fogel et al. [43] reported an increase of sigma power during Stage 2 sleep and slow wave sleep following periods of procedural learning. Unexpectedly, we found a significant decrease in sigma power (12–16 Hz) in frontal and central electrodes during Stage 2 sleep following training onset with the belt whereas control participants showed no significant changes in the power analysis. In line with our result, however, Campus et al. [75] observed a similar effect in an experiment on sensory substitution involving supramodal mental mapping both in blind and sighted subjects. They specifically explored the lower beta frequency band, due to its role in short-term memory [76] and complex associative functions [77]. In their study in both blind and sighted subjects, low beta power decreased after active exploration of a virtual environment with a sensory substitution device. This decrease was suggested to be associated with motor programming [75]. Additionally, the power decrease after active exploration of the environment was proposed to be caused by increased sensorimotor processing after tactile stimulation [78,79]. Therefore, the observed decrease in sigma power during sleep in the present experiment might indicate increased sensorimotor processing and motor programming in the early training with the *feelSpace* belt.

### Training and involvement of cortical areas

The fMRI measurements during virtual navigation showed that training with the *feelSpace* belt influences activation of sensory and higher motor brain regions. We observed a differential activation in the primary and secondary somatosensory cortex and the insula, structures known to be involved in sensory processing [80–83]. Additionally, we found a significant activation difference in the superior temporal gyrus (STG) and in the right posterior parietal cortex (PPC). In the navigation and control condition participants received tactile belt information and visual cues in the form of optic flow to solve the task. However, they lacked other sensory information like vestibular or kinesthetic information. Therefore, we assume that the activation of early sensory areas reflects the processing of the tactile belt signal. The STG is known to be a polysensory spatial area in which multimodal sensory input converges into higher order spatial representations [84]. We hypothesize therefore, that the finding of an activation of the STG indicates integration of tactile signals provided by the belt and visual information [85]. Additionally, we found an activation of the PPC that contributes to sensory-motor functions and transformations [86], somatosensory and motor integration [87,88], and spatial attention [89,90]. Taken together, our fMRI results revealed a differential activation of brain areas known to be involved in sensory processing and in sensorimotor integration.

We hypothesized that after a training period that is sufficient to induce observable effects with the *feelSpace* belt also changes in brain activation would be observable. In our experiments, we observed a significant reduction of activation in SMA and premotor cortex after the training period. This is in line with previous work on learning to control a brain-computer interface [91] that found a decrease of initially high activation in prefrontal cortex, premotor cortex, and PPC once participants learned to master the brain-computer interface. A decrease of activation in the premotor cortex has also been found as an effect of procedural learning [92], in line with the results obtained by the sleep EEG described above. Furthermore, the two-way interaction comparing the belt signal and the time of measurement (date*belt), revealed a change in the activation pattern. These changes were especially marked in S2, where we found a reduction of activation differences after the training period. This suggests that belt training is accompanied by a change of how the belt’s signal is processed. In the literature, the effects of training on changes in neural functions are heterogeneous: Practice-related activation changes may result in an increase or a decrease in activation of involved brain areas as well as in a reorganization of activated areas [93,94]. Activation decrease is a common finding in examining task practice. The main mechanism, which is proposed to underlie activation decreases, is an increase in neural efficiency, sometimes called sparsification [95,96], which is suggested to be the cognitive consequence of greater skills at applying the initial strategy [97]. A reduction of premotor activity is also compatible with the skilled attention hypothesis [98], which proposes that the control of attentional processes within a domain becomes an aspect of the skill and less volitional as the procedural repertoire becomes more flexible and robust against distortions, leading to an improved mastery of the domain. Therefore, our finding of a decreased activation in sensory and in higher motor areas after the belt training period might indicate a training induced increase in neural efficiency.

### The influence of “navigation” onto cortical activation pattern

As the *feelSpace* belt delivers continuous information about orientation in space, we hypothesized that this would also modulate activity in brain areas known to be involved in navigation. To address this question, the fMRI measurements compared a virtual homing task and a control task in close analogy to the task in Wolbers et al. [62]. In this comparison our results revealed a higher activation in the homing than the control task in a large sensorimotor network including S1, Insula, PPC, MST, premotor cortex, and SMA as well as in the Cerebellum and the caudate nucleus. As known from previous work, MST is involved in extracting heading information from optic flow [99,100], reflecting the optic flow in the performed homing task. SMA is known to be involved in the control, planning, initiation, and execution of movements [101–103]. The premotor cortex is mainly involved in sensory predictions and polymodal motion processing [63], as well as understanding of motor events [104]. As the participants did not move in the scanner, the activation of these motor areas presumably reflects the imagined movements in the virtual navigation task. The cerebellum was reported to be involved in predictions about sensory consequences of actions [105]. Furthermore, previous studies found evidence for the involvement of the Cerebellum in navigation [106]. It was reported that the caudate nucleus is also involved in navigation [107], especially during route following [108] and way finding [109]. In the two-way interaction comparing *date and task* we found significant differential activation in the cerebellum and the caudate nucleus. In the cerebellum, we only observed after the training a significantly higher activation in the *homing* than in the *control* task. The increased cerebellar activation during the homing task could thus be understood as a reflection of the necessity of predicting the sensory outcome of the imagined movements in the virtual environment. In the caudate nucleus we found only in the homing task a higher activation before compared to after training. These results reveal differences in the activation pattern in the cerebellum and caudate nucleus for the homing and the control task depending on before or after the training. Unexpectedly, we found a significant activation difference in the right hippocampus between control and belt wearing participants with a higher activation in the control group. The hippocampus is a region that is often involved in navigation tasks [45,110,111]. Hippocampal activation is usually investigated and observed in connection with memory tasks. Specifically, hippocampal activation is found in tasks when memory retrieval is relevant for forming cognitive maps [111,112], navigating successfully in learned environments [45,113], and when tasks include landmarks [114]. Evaluating qualitative reports of subjective experiences of the seven weeks training period of belt wearing and control participants Kaspar et al. [30] found that the navigation strategies that were used while navigating in natural environment differed between groups. The belt wearing participants reported to more and more rely on the *feelSpace* belt’s information for navigation whereas all control participants reported to use landmarks and most also city maps. Furthermore, belt wearing participants reported that using the belt enabled a more intuitive navigation with less cognitive effort [30]. These differences in navigation strategies reported by the belt wearing and control participants might relate to the differential activation observed in the hippocampus. Also, Wolbers et al. [62] found right hippocampal activation in a similar task to ours only in correlation with pointing accuracy, which they suggested to show strong engagement to be necessary for accurate updating. Taken together, these data demonstrate the differential activation of cortical areas related to spatial navigation.

### Behavioral evaluations

We assessed behavioral changes induced by training with the *feelSpace* belt by measuring participants’ performance in a complex homing paradigm. With our task design, in comparison to the classical triangle completion task, we aimed at task conditions that were planned to induce continuous updating of a homing vector against a natural human tendency to solve homing via survey reconstruction [115]. To make it harder to solve the homing task by means of cognitive reasoning we additionally added a memory task for increased cognitive load. Here, improvement of homing performance after the training period with the *feelSpace* belt did not reach significance. A number of factors might contribute to this negative result. First, even though Wiener and Mallot [116] demonstrated in a visual speeded point-to-origin task that increasing path complexity does not necessarily negatively influence path integration abilities, path complexity might have an influence on performance in a real world navigation task. This is in line with the predictions of most common path integration models for humans [115]. Indeed, a previous study investigating a homing task using the *feelSpace* belt [28] found an improvement of task performance after the training period with less complex polygons. Second, recent research on spatial attention of vision and haptics indicate shared attentional resources [117] and a task dependence of visuotactile processing [118] but no influence of attentional resources on optimal visuotactile integration. These findings suggest that our dual task design of path integration combined with a number memory task have not limited the integration of tactile signals in this complex task. Third, we have to consider that the spatial dimensions of the homing task were still rather small compared to natural settings. The polygons were of a length of 19–22 m and path completion took less than a minute. This is a scale where signals supplied by the vestibular system are still reliable. Thus, in the absence of belt signals participants might rely on the vestibular information. To address this shortcoming, we designed a large-scale pointing task when studying a congenitally blind subject [31] that was then also used in a study with a late-blind participant [29]. The results revealed significant performance improvement for the late-blind participant after the training period with the *feelSpace* belt [29]. The results with a congenitally blind subject indicate that performance improvements with the *feelSpace* belt depend on the navigation strategy spontaneously employed by the perceiver, and can be largely enhanced by training a strategy suited for the information of the belt. The evaluation of the large scale pointing study with sighted participants is ongoing. Therefore, in the present study we have to consider that the behavioral task as part of the overall study was not optimized to test for training-induced behavioral changes with the *feelSpace* belt.

### Perceptual changes

As perceptual changes are an important aspect of SMC theory we thoroughly evaluated subjective experiences of participants in the course of the seven-week training period. Here, we report changes in perception following the training and the influence of the training duration comparing belt wearing and control participants. In the belt wearing group, we found a significant increase throughout the training period of subjective ratings concerning *space perception*, *trust in navigational ability*, *tactile belt perception*, and (the inverted scale of) *conscious belt perception*. This indicates, in line with previous studies investigating sensory substitution [21,22], that the longer participants train the more detailed perceptual changes occur. Importantly, the active exploration and training with the sensory augmentation device, as used in our study, to improve the richness of the perceptual changes is supported by previous studies using sensory substitution [25,119,120]. In contrast to belt wearing participants, control participants actively training their orientation did not report systematic changes over the training period. Evaluating the development of a new perception of space using the feelSpace belt Kaspar et al. [30] found that after seven weeks of training eight out of nine belt wearing participants stated to have developed a new spatial perception. In contrast, no control participant reported the development of a new space perception. Our finding of a quantitative increase of rated perceptual changes over time is supplemented by the qualitative analysis of subjective reports of perceptual changes [30]. The main focus of reported changes in space perception concerned spatial relations between self and cardinal directions, self and objects, between objects, alignment of objects towards cardinal directions as a new feature of the objects, and updating and enlargement of mental maps, which then provided a basis for spatial orientation [30]. Thus, the increase of quantitative perceptual changes over time and qualitative perceptual changes when training with the *feelSpace* belt are compatible with the theory of sensorimotor contingencies.

### Considerations on study design

Our experimental design is a first step of a series of increasingly refined tests using sensory augmentation. For example, in the present work we compare belt wearing participants with controls who do not have any directional information and do not wear any belt as such. This comparison was driven by the aim to have a control group that moves equally in natural environment and have the belt as the defined difference. As the SMC theory states the importance of action for developing new SMCs a possible control would also be a passive training condition. In fact, a recent study on integration of kinesthetic and vestibular information by EEG in a virtual reality environment reported significant differences in cortical processing between active and passive conditions [121]. To apply such techniques in the context of sensory augmentation is an important next step.

As we have a very complex and demanding study design including a series of different experiments, we had to find a balance between desirable amount of data and the demand on participants and length of the whole project. This lead to a training duration of seven weeks for which a former study [28] had shown significant perceptual and behavioral changes. Previous studies examining sensory substitution [21–23] showed that some perceptual aspects of the substituted stimulus were learned in a very short time whereas a prolonged training with the device developed a more detailed perception. Ward and Meijer [22] investigated late blind subjects with an auditory to vision substitution device and even after several years of daily use still observed perceptual changes. In line with this finding our subjective data indicate that the learning process did not asymptote within seven weeks. Thus we hypothesize that a prolonged training duration would lead to more pronounced training effects. Furthermore, the complex study design also influenced the number of participants resulting in small unequal groups. Thus, our results give a first report of effects, which have to be explored in detail with a large-scale study and an even longer training duration.

Out of ethic considerations we performed our study with adult participants. As previous studies showed that the human brain is plastic throughout the life span [12,14], we hypothesized that training with the sensory augmentation device would lead to observable changes in the brain. One mechanism to induce brain plasticity is crossmodal and sensorimotor activation, which could be observed, e.g., in sighted subjects through visual and haptic object recognition [122,123], in blind subjects in auditory verb-generation [124], in speech comprehension [125], and in Braille reading experiments [126,127] and in sensorimotor learning [128]. Related to the theory of the critical period [129], where brain plasticity is assumed to be restricted to a crucial time gap in early life, several research groups investigated brain plasticity in blind subjects who were born blind or lost sight early or late in life [130–132]. These groups suggest, in accordance, that there is a difference of compensatory plasticity in congenitally and late blind subjects with a susceptible period seemingly before adolescence (in these studies between 12 and 16 years of age). Therefore, we hypothesize that even though our results suggest procedural learning and changes in brain activation following the training with the feelSpace belt in our adult participants that these changes would be even more marked when training with sensory augmentation before adolescence.

### Conclusion

With our study design we introduced a practical possibility to investigate the development of new sensorimotor contingencies by means of sensory augmentation. The measurements were designed to test predictions of SMC theory [10,55]. Specifically, compatible with a procedural learning process, in the early training phase we found indications for increased sensorimotor processing and motor programming in sleep EEG recordings. Investigating brain activity with fMRI revealed an involvement of a large sensorimotor network and areas that are known to participate in navigation. Perceptual changes increased continuously with training duration, thus supporting the notion of SMCs, which postulates that mastery of sensorimotor contingencies is constitutive of conscious perception [10,55]. The present study motivates us to further investigate the grounding of conscious perception in the concept of SMCs and the approach of embodied cognition. Our findings using sensory augmentation in a real world environment might also encourage practical use in the field of sensory substitution.

## Materials and Methods

Our study complied with Helsinki Declaration guidelines and was approved by the ethics committee of the University Osnabrück. All participants were extensively briefed in a dedicated meeting and provided informed, written consent before participating.

### The *feelSpace* belt

The *feelSpace* belt, in its first version designed by Nagel et al. [28], is a sensory augmentation device that supplies information about magnetic north as vibrotactile information around the waist. For this, a belt was equipped with an electronic compass, a set of 13 vibrotactile actuators, battery packs, and a control unit that always activates the actuator pointing north. Specifically, only the one northernmost element is vibrating, as participants feel irritated when more than one vibration element is active [28].

Here, we developed a special non-magnetic variant of the *feelSpace* belt for use in magnetic-resonance (MR) environments using piezo-ceramic actuators. As we are interested in isolating neural activity selectively related to the directional information conveyed in the tactile signal, it is essential to keep the vibrotactile signal proper identical between daily training and fMRI-testing situations. We therefore also developed a set of portable piezo-ceramic *feelSpace* belts for everyday use during the training. These portable belts accompany the MRI compatible version and utilize the same piezo-ceramic actuators and piezo-driving signals. Hence, our study on sensory augmentation provides the identical tactile stimulation in training and in all tests, including fMRI measurements [31,58].

**Fig 6:**
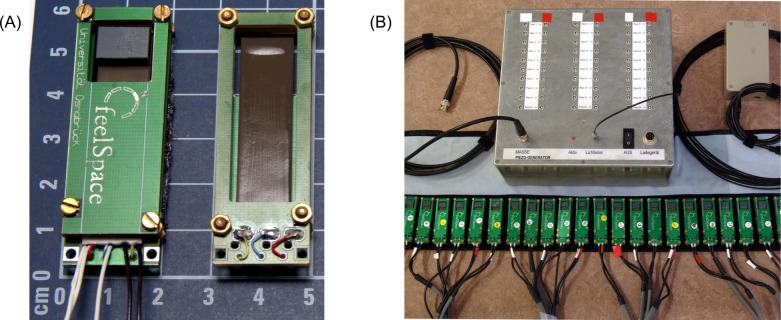
Details of the *feelSpace* belt. (A) Individual vibration elements feature casing, strain relief of power supply, piezo ceramic bending actuators, and a stamp. (B) The MRI compatible belt is connected by 60 coaxial cables to a filter box, which in turn is connected to the scanner room’s Faraday shield. Figures taken from Keyser [58] and under Creative Commons CC-BY-3 from Schumann [31].

Both belt variants are made of a modular core unit that entails 30 non-magnetic piezo-ceramic bending actuators as vibration devices (PL140.10, Physikinstrumente GmbH & Co. KG, Karlsruhe, Germany). Thus, we more than doubled the spatial resolution of the original belt. Piezo-ceramic actuators are MR compatible [133,134] and have been used previously in fMRI experiments [135]. To provide physical stability for the highly fragile ceramics, and a means of fixation to the belt, each actuator is placed in a custom-made housing build from resistant PCB material that covers most of the actuator and absorbs moderate shocks. A small plastic plate glued at one end of the piezo ceramics provides the surface contact of the vibrotactile stimulation to the skin (Fig 6A). For further insulation, piezo-ceramics and electrical contacts are individually covered with an acrylic thin film coating that also provides water-resistance. Each actuator housing is mounted individually to the belt via a Velcro strip so that the actuators can be distributed evenly for varying sizes of the belt.

In addition to MRI compatibility, our re-design of the *feelSpace* belt entails a number of further improvements over the original version. Electro-magnetic actuators used in the previous belt have a high-latency onset of activation of typically 200ms, while piezo actuators used here achieve a fast activation onset below 10ms. This allows an almost instantaneous change of the tactile signal without noticeable response latencies. Both control logic and compass were selected to utilize this high speed. Thus, our piezo actuators operate at a time scale that is close to other sensory information such as vestibular signals, proprioception or vision [136]. Second, piezo-ceramic actuators allow a precise design of the tactile vibration signal. Vibration frequency was set to about 178 Hz as an optimal sensitivity to tactile vibrations is achieved at frequencies between 150 and 300 Hz [137]. Lastly, we drastically increased the number of actuators to a total of 30. The placement of the actuators now is fine-graded relative to the discrimination thresholds of skin surface around the waist [138], and the refined angular resolution of the novel belts requires only a small turn of 12° degree for switching to an adjacent actuator and change in sensation. Hence, the piezo-ceramic belts provide a smooth and low-latency sensation of a counter-rotational movement when turning around the longitudinal body axis with the belt.

To prevent electromagnetic disturbances, in the MRI-compatible version of the belt each actuator is interfaced individually by a shielded coaxial cable of 5 m length connected to the control electronics via low-pass filters and high quality LEMO connectors. The control electronics is kept at a maximum distance to the scanner coil and electrically isolated from the stimulus computer in the experimentation room by an optical cable. For hardware shielding a 30-channel filter-chain system is contained in three separate fully closed aluminum boxes of 10 filters each, which are placed together with an optical control board, a modular generator for the piezo-driving signal, and six 0.8 Ah sealed lead-acid batteries in a further enclosing aluminum case. The modular piezo-driving generator uses a dedicated microprocessor to generate a 178 Hz square wave signal that switches the 22 V DC supplied by lead acid batteries as the piezo-driving signal. The driving signal in turn is directed to an individual piezo bender via optical relays, which also provide optical isolation between actuator and power supply. For electrical shielding, each piezo actuator channel is filtered with 60 dB low-pass filters at 120 MHz, the approximate resonance frequency of protons in a magnetic field at 3 T. The filters connect the piezo ground to the enclosing aluminum case, which is in turn connected with low-impedance to the Faraday cage of the scanning room to dissipate any high-frequency energy that might have been induced. Dedicated tests showed that the fMRI *feelSpace* belt operates without noticeable influences on the MRI signal.

In the portable belt, the actuator core unit of the belt additionally contains an electric compass, a central control unit, power supply and management, and the identical modular piezo-driving signal generator from the MRI variant of the belt. The compass (3DM-DX-3-25, Microstrain) integrates 3-dimensional accelerometer and gyrometer inertial sensors with a magnetometer signal. It is one of the smallest orientation sensors of its kind and provides reliable and highly accurate directional information during the whole range of human movements. A custom made, Arduino-based control logic reads the compass signal and instructs the 30-channel piezo-driving board to switch on the actuator that points north. A GPS unit and data logging on a micro SD card allow quantifying the movement activity as well as the variety and regularity of the environments explored during the training. High-capacity lithium batteries provide daylong usage of the belts. A power management circuit transforms the output voltage of the batteries to the piezo driving voltage and prevents critical deep discharge of the lithium batteries. Hence, even using sophisticated piezo-ceramics as tactile actuators, the belt needs only to be charged during the night and can be used with ease during a full day.

A flexible and water-resistant fabric covers all actuators and electronic parts to provide comfortable use in both variants of the piezo-ceramic belts. Lithium batteries are extra separately encased in fireproof sacks for enhanced security of the participants.

### Sleep EEG Measurements

For the part of the project that involves the sleep EEG, an initial interview was conducted to exclude participants with atypical sleep patterns (shifted sleep times outside the approximate hours between 11:00 pm and 7:00 am, difficulties in falling asleep, and nocturnal awakening). One participant (BWP 8) was excluded from all sleep EEG analyses because of poor sleep quality (more than 20% of awake and 20% of stage 1 sleep in the EEG data throughout the baseline and first test night, and repeated awakening during the nights reported by the experimenter in the laboratory). Therefore, only eight participants who wore the belt (five males and three females) and five control participants were included in further analysis. All sleep measurements took place in the EEG laboratory at the Neurobiopsychology Group, University of Osnabrück. Before the start of the experiment, each participant had a 1.5h adaptation and screening nap. The nap served for participants to get familiarized with the procedure of EEG setup preparation, the sleep facility, and to reduce the “first night effect” [139]. The nap was also used as a screening procedure to exclude participants from future involvement in the experiment due to problems with falling asleep in unusual or unknown environments, and any types of sleep disturbances. All participants spent four nights in the laboratory, including one baseline night. Time spent in bed was restricted to eight hours and was fixed from 11:00 pm to 7:00 am for every participant. The first night was used to collect the baseline EEG for further within subject comparison of subsequent recordings. Further nights were planned in order to obtain data over a longer period of the learning process. After this learning period, the data were used to identify effects of using the belt over time. As most noticeable effects of learning were expected in the beginning of training, recordings were scheduled tighter during the early training period. The most noticeable effect was expected to occur after the belt on-set and following early training. Thus, participants spent the first and fourth nights in the sleep laboratory after the beginning of the training. The last night at the end of the training was used as the post-training measurement. Before every sleep onset in the laboratory, a sleep-wake questionnaire was used to screen participants for sleep quality and quantity, sleepiness, caffeine, nicotine, and alcohol consumption.

Sleep EEG was recorded with a Ready-to-use EEG Recording Cap for 19/21 Channels by Easy Cap™. The electrode arrangement was based on the international 10–20 system for electrode placement. Two reference electrodes were placed on the skin above the mastoid bones, ground placed at FPZ. EEG, EMG, and EOG signals were sampled at 500 Hz and acquired continuously during the night. Impedances were kept below 5 kOhm at the beginning of each recording. In addition to the EEG-recording, we used a night-vision camera in a sleeping room to control participants for body movements during the night.

Four independent judges scored sleep EEG recordings in 30 s epochs according to the standard criteria (AASM, 2007). All judges established scoring reliability among each other with above 90% agreement. EEG signals from all 10 channels were filtered between 0.5 Hz and 35 Hz, bad channels were rejected and signals were average referenced. To determine if training with the belt had an effect on sleep architecture, we compared the duration of time spent in specific stages of sleep (stage 2, SWS, and REM) from baseline to the test nights. Time spent in every sleep stage was calculated as a fraction of the whole night duration. A one-tailed paired-sample permutation test with 10^5^-sample size on the sleep stages duration was applied for within- and between-group pairwise comparisons.

For further power spectral analysis, EEG recordings were visually inspected for segments containing artifacts, which were then excluded from all quantitative analyses. We performed an all-night spectral analysis on the same 30 s epochs for which sleep stages had been determined. Within each artifact-free epoch, spectral power was calculated using the routine Fast Fourier Transformation (FFT) technique for 4 electrode derivations (F3, F4, C3 and C4). Power spectra were estimated by means of the Welch method (50% overlapping with 4 s Hamming windows). We further focused our analysis on three sleep stages (SWS, REM, and stage 2 sleep) and computed average spectral density for three frequency ranges being representative for each of these sleep stages (delta: 0.5–4 Hz, theta: 4–8 Hz and sigma: 12–16 Hz respectively). Mean values of log transformed absolute power values for each of these frequency bands were analyzed separately with a mixed-measures ANOVA with experimental nights and electrodes derivations as within subject factors and group (belt wearing participants vs. control participants) as a between subject factor. Each significant finding was followed up with a paired t-test.

### FMRI Measurements

During fMRI measurements participants viewed a minimalistic virtual environment from a first-person perspective on a computer screen while the belt provided related tactile signals. The experiments involved *experimental participants* and *controls* performing a *homing* and a *control task* with belt *on* and *off*, *before* and *after* training. This resulted in a complete 2 x 2 x 2 x 2 design.

**Fig 7:**
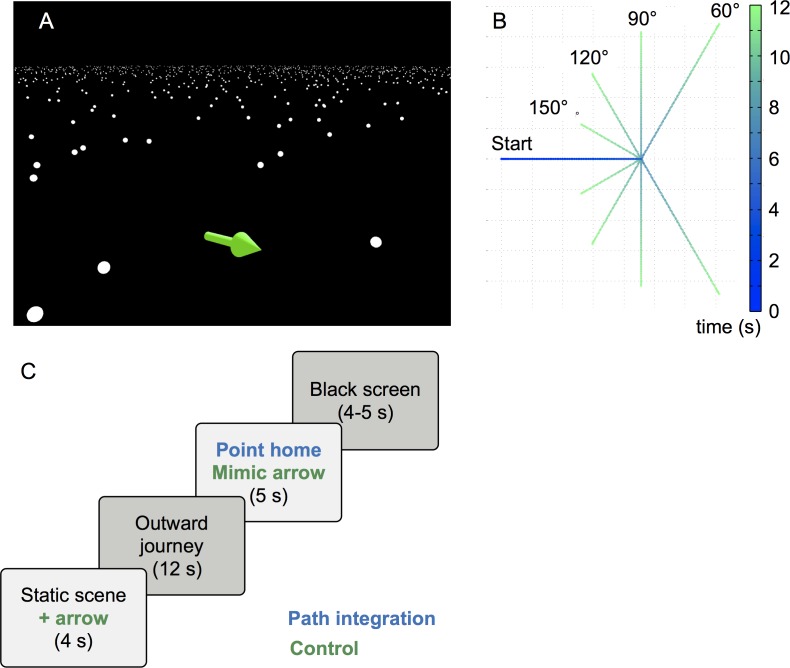
Schematics of the fMRI path integration task. (A) Screenshot of the virtual environment and the response arrow. A surface texture of minimal-lifetime dots provides optic flow during outward journeys. (B) Triangles used in the outward journey traveld by subjects. The first segment of each triangle had constant velocity and a duration of 4s. The length of the second segment was adjusted according to the prior turning angle to ensure a total travel duration of 12s in all trials. (C) Flow of the conditions. During path integration, subjects should point the response arrow back to the starting location. During control, subjects should replicated the angle of an arrow additionally presented at the first static scene of control trials. Figures taken from Keyser [58] and under Creative Commons CC-BY-3 from Schumann [31].

We used the paradigm of Wolbers et al. [62] to assess modulation of activity of brain areas involved in path integration. Participants passively traveled along two legs of a triangle and finally pointed towards the starting location with an MRI-compatible joystick (Fig 7). Instead of remembering the starting location, during control trials participants were asked to memorize the ego-centric direction of an arrow presented before the onset of the trial, and to point towards the direction of this arrow again in ego-centric coordinates after traveling along the second leg. That is, in the control condition participants experienced identical visual and belt stimulation and also performed an identical motor task, but were not asked to take into account the changes in the heading direction that are necessary in the homing condition.

Participants saw a minimal visual environment from a first person perspective that provided only optic flow by a star field composed of limited-lifetime dots (Fig 7A). Virtual motion was passive to avoid confounding motor activations and to ensure identical travel durations in each trial and participant. We used eight outbound paths across all conditions. Paths were comprised of one intermediate rotation and two translations. To allow for identical onsets of the rotation period, the length of the first translation was kept constant (at 8.5 m). Intermediate rotations differed in turning direction (left, right) and turning angle (60°, 90°, 120°, 150°). Since path integration in virtual environments is most accurate when displacement velocities resemble those of natural locomotion [140], we used a speed of moderate walking for the translation (maximum speed 2 m/s) and the rotation (maximum speed 40°/s). Sequences of translation and rotation followed the same trapezoid velocity profile with linear increases and decreases of velocity. The plateau of the trapezoid velocity profile changed according to the length of the translation or rotation angle. To also keep total travel times constant over all trials, the lengths of the second translation were adjusted depending on the angle of the intermediate rotation, i.e., the second leg was shorter for trials with longer rotations, leading to a constant trial duration of 12 s (Fig 7B). Hence all trials had identical rotation onset times as well as total durations.

The overall study design contained a pre scanning training session of the homing paradigm outside the scanner, and the fMRI version of the homing task for the actual measurements. This allowed us to familiarize participants with the homing paradigm prior to the fMRI measurements. To minimize learning effects during the scanning further, participants also received training in virtual triangle completion in the horizontal body position outside the scanner before the actual measurements. Training sessions used a different set of triangles with different turning angles as those of the fMRI experiment. During training, trial responses were followed by instant feedback, i.e., by providing an arrow that indicated the correct direction towards the origin. Immediately before the actual measurements, participants received one additional training session of 16 trials within the fMRI environment prior to the experimental session proper. Each path was repeated five times with and five times without the belt information in pseudo-randomized order and with control of sequential effects. This yielded a total of 160 trials (80 experimental, 80 control). In the horizontal position, virtual north was arbitrarily but consistently over all trials defined in a virtual “magnetic field” displayed as if subjects had a vertical position. During travels on outbound paths, the belt signal was continually updated with respect to the direction of virtual north from the current position in the virtual space. To prepare subjects, each trial started with a static presentation of the virtual environment for 4 s that indicated the condition, followed by the outward journey that always lasted 12 s. At the endpoint of the second translation, subjects used an MR-compatible joystick to point toward the origin of the travel within a 5 s interval (Fig 7C). Pointing responses were recorded when joystick deflection exceeded 80% of maximal deflection. During inter-trial intervals, a black screen was presented randomly for 4 or 5 s. This yielded a net scanning time of 40 minutes for the experimental and the control conditions, respectively.

Visual path integration involves both the processing of self-motion cues, as well as a working memory component for changes in distance and direction from the starting point. To isolate processes of path integration, a control task was necessary that provided identical visual stimulation and motor responses, a working memory component unrelated to the path, as well as identical belt stimulation. In the final control session, subjects traveled along the same 80 paths as in the experiments. However, during the initial 4 s starting period, an arrow was presented in parallel to the ground plane and subjects were asked to remember its direction. At the endpoint of the translation, subjects had to point into the direction of the arrow shown in the beginning of the trial (Fig 7C). With the belt, path integration additionally requires somatosensory processing of the belt stimulus. Therefore, subjects also performed the control condition with and without the belt. In this control condition the global orientation aspect of the belt signal is irrelevant for the task while the tactile aspect of the belt signal is preserved. Control tasks were recorded in separate sessions to minimize the possibility that subjects engage in path integration during the control task. In summary, control trials provided identical visual and somatosensory stimulation, an identical motor response, as well as a working memory component that is unrelated to the travel path but did not require subjects to integrate the path.

For pre-processing of each MRI-run, we discarded the first five scans from further processing in order to reject remaining tissue saturation effects. The remaining scans were slice-time corrected, spatially realigned to the first volume, and normalized into MNI space using the segmented and structural image. Finally, they were spatially smoothed with an isotropic Gaussian kernel of 8mm FWHM.

In the first level design, in accordance with Wolbers et al. [62], we separately modeled the trials’ 12s outbound path and 5s response periods as boxcar functions, which were convolved by SPM’s canonical hemodynamic response function. A high pass filter was applied to remove baseline drifts. Each participant’s data from the two measurement dates (*pre*, *post*) were modeled separately. Per date and participant, two MRI runs were recorded, interrupted by short breaks after 25min. Both runs were modeled within a single GLM, as two distinct sessions with individual intercept regressors. The regressors for outbound paths and response periods were separately defined for each level of the factors of within-subject factors *date*, *belt*, and *task*. Outbound paths with the same absolute turn values of 60°, 90°, 120° and150° were collapsed into the same regressors. Trials in which participants failed to respond within the response interval of 5s were defined as a separate regressor and excluded from analysis. Only the outbound path regressors were used for 2^nd^ level analyses by application of the appropriate contrasts. In order to account for all our independent factors of interest (*group*, *date*, *belt*, *task*) in a statistically sound way, contrasts were derived from the first-level designs. Dependent on the nature of first-level statistic, these contrast images were then subjected to an appropriate second-level design using the GLMFlex extension. Specific effects were tested with the appropriate linear contrasts of the 1st level parameter estimates, and the resulting images were subsequently entered into a random effects analysis. We tested for main effects of the belt signal (*belt*), of homing (*task*), of control and belt wearing participants (*group*), as well as before and after training (*date*). The goal of this analysis was to assess the effects of all four independent factors of interest (*group*, *date*, *belt*, *task)* using a mixed-effects design. Input to the second level consisted of four contrast images from each participant’s *pre training* and *post training* first-level GLMs: Within each first-level model and each level of *belt*, we subtracted *control task* from *path integration*.

### Homing Task Measurement

We designed an innovative homing paradigm as an alternative to the conventional triangle completion task, consisting of eight carefully crafted, complex, curvy paths. Unlike most animals, humans have a tendency to solve a homing task on simple figures via survey reconstruction. Survey reconstruction is based on a segmentation of the path into edges and angels rather than by updating of the homing vector [115]. Hence, the design of the figures was guided by the intention to trigger continuous updating in a paradigm that is less effectively resolvable by means of a configural strategy. For this purpose, complex figures without easily separable edges (= no ′′landmarks′′) comprising less frequent rotation angles (e.g., no 90° angles) are most suitable as these are hard to visualize mentally. While solving the homing task, participants additionally had to memorize numbers as a cognitive load.

The paths are based on non-orthogonal polygons: two rectangles, two pentagons, two hexagons, and two heptagons. None of the polygons included crossing-overs. Half of the figures were traversed clockwise, the other half counter-clockwise, with varying homing angles for each shape. The overall paths were between 15 and 18 m, not including the homing distance. As we have already a complex study design (*date*, *belt*, and *group*) on unusually difficult polygons we kept the homing segment constant in length over all figures (= 4 meters) even though in classical navigation studies, both the homing angle *and* the length of the homing segment are varied (for conventional practice, see for example [37]). Additionally, pretests showed that participants did not realize that the length was actually always the same. Wiener and Mallot [116] demonstrated that, contrary to the predictions of most common path integration models [115], increasing path complexity did not negatively influence path integration abilities in a speeded point-to-origin task.

Participants performed the homing task before the start of training (with or without the belt respectively) and again in the last week of training. All sessions were performed indoors, in the wind and sun shielded environment of a large hall. They consisted of two exercising runs and the subsequent actual measurements. During the whole session, participants were blindfolded and wore earplugs to eliminate both visual and auditory cues. Before conducting the actual homing measurements, participants underwent two trainings to minimize learning and habituation effects. This was achieved by familiarizing the participant with his or her environment, setting (e.g., moving freely while being blindfolded), and task. In total we collected 32 homing vectors per participant, i.e., one for each condition (*pre* vs. *post*; *belt on* vs. *belt off*) for each figure.

The general process of the homing task was similar to procedures described in classical homing studies: the experimenter guided the blindfolded participant from the origin along a path by means of the wooden handle bar, released him at the homing point, where the participant hereupon turned into the direction of origin and walked back using the shortest way. Meanwhile, the time participants needed to decide in which direction to go was recorded, until they left an area with a radius of about 40 cm around the homing point.

Additionally our participants had to solve a cognitive load task while performing the homing task. Standing at the starting point, the investigator read out a list of four numbers the participant was supposed to keep in mind during the homing task. The numbers were in a range between 1 and 40, resulting in sequences comprising two single-digit and two multi-digit numbers. When participants reached the assumed starting position, they recited the sequence of numbers.

### Subjective evaluations

To evaluate subjective experiences, we designed daily and weekly questionnaires (see also [29,30]). To assess qualitative experiences and quantitative estimations of changes we used a mixed method approach (e.g. [57]). Therefore, both questionnaires contained qualitative, open-ended questions and quantitative 5-point Likert items. The daily questionnaire includes items measuring the kind and duration of activities participants performed during their daily training with and without the belt. Additionally, in the daily questionnaire sleep quality of the last night and participants’ state of health, their happiness, alertness, calmness, and listlessness were assessed. Furthermore, participants were asked to write down all experiences they had during the last day. For the belt wearing questionnaire items relating to the belt were included. These items asked for how long participants wore the belt and whether technical problems occurred and if so of what kind the technical problems were. Participants were asked to report problems with the belt directly, so that a longer training outage could be avoided. The weekly questionnaire was designed to get insights into possible changes for the aspects of space perception and belt perception, and about influences of training with and without the belt, respectively. Those topics were merged into quantitative items. To get an explicit statement whether a new sense of space perception developed we included a special single item that had to be answered with yes or no in both questionnaires. These items were complemented with open-ended questions concerning changes in the perception of the belt signal (only belt questionnaire), changes of the mental map and changes in space perception (belt and control questionnaire). The questionnaires for the control participants were created in close analogy to the belt wearing questionnaire with the exclusion of the special belt signal items. Instead of reporting their experiences “with the belt”, control participants were asked for their experiences after “training their orientation”. In the control items the phrasing of the items was the same except substituting “with the belt” with “orientation training” in the control questions. Participants filled in the daily questionnaire each day at home. These questionnaires were collected weekly at a meeting in the laboratory where participants also completed the questionnaire and a weekly supplementary interview.

We additionally evaluated before the start of the training period the German version of the NEO-FFI [68] and the ACS-90 [69] to asses relevant personality traits and the “Fragebogen Räumliche Strategien” (FRS) to asses navigational behavior. The FRS was again evaluated directly after the end of the training ended and two month later. For the belt wearing group we weekly measured the AttrakDiff2 [71] Questionnaire to assess the *feelSpace* belt. These results as well as the results of the daily questionnaire and all qualitative data have been published in Kaspar et al. [30].

## References

[1] Engel AK, Maye A, Kurthen M, König P. Where’s the action? The pragmatic turn in cognitive science. Trends Cogn Sci. 2013;17:202–9. 10.1016/j.tics.2013.03.006 23608361

[2] Marr D. Vision: A Computational Investigation into the Human Representation and Processing of Visual Information. New York, US: Freeman; 1982.

[3] Clark A. An embodied cognitive science? Trends Cogn Sci. 1999;3:345–51. 10461197 10.1016/s1364-6613(99)01361-3

[4] Varela FJ, Thompson E, Rosch E. The Embodied Mind: Cognitive science and human experience Cambridge, MA: The MIT Press; 1991.

[5] Wilson M. Six views of embodied cognition. Psychon Bull Rev. 2002;9:625–36. 12613670 10.3758/bf03196322

[6] Barsalou LW. Grounded cognition. Annu Rev Psychol. 2008;59:617–45. 10.1146/annurev.psych.59.103006.093639 17705682

[7] König P, Wilming N, Kaspar K, Nagel SK, Onat S. Predictions in the light of your own action repertoire as a general computational principle. Behav Brain Sci. 2013;36:219–20. 10.1017/S0140525X12002294 23663324

[8] Ziemke T. What’s that Thing Called Embodiment? Proceedings of the 25th Annual meeting of the Cognitive Science Society. Mahwah, NJ: Erlbaum, Lawrence; 2003;1305–10.

[9] Mangen A, Velay J. Digitizing literacy: reflections on the haptics of writing. Adv Haptics. 2010;385–403.

[10] O’Regan K, Noe A. A sensorimotor account of vision and visual consciousness. Behav Brain Sci. 2001;24:939–1031. 12239892 10.1017/s0140525x01000115

[11] Clark A, Tower DH, Square G. Vision as Dance? Three Challenges for Sensorimotor Contingency Theory 1. Psyche. 2006;12:1–10.

[12] Pascual-Leone A, Amedi A, Fregni F, Merabet LB. The Plastic Human Brain Cortex. Annu Rev Neurosci. 2005;28:377–401. 10.1146/annurev.neuro.27.070203.144216 16022601

[13] Amedi A, Stern WM, Camprodon JA, Bermpohl F, Merabet L, Rotman S, et al Shape conveyed by visual-to-auditory sensory substitution activates the lateral occipital complex. Nat Neurosci. 2007;10:687–9. 10.1038/nn1912 17515898

[14] Proulx MJ, Ptito M, Amedi A. Multisensory integration, sensory substitution and visual rehabilitation. Neurosci Biobehav Rev. Elsevier Ltd; 2014;41:1–2. 10.1016/j.neubiorev.2014.03.004 24759484

[15] Ptito M, Kupers R, Lomber S, Pietrini P. Sensory deprivation and brain plasticity. Neural Plast. 2012;2012. 10.1155/2012/810370 PMC3474285 23094163

[16] Bach-y-Rita P, Collins CC, Saunders F a, White B, Scadden L. Vision substitution by tactile image projection. Nature. 1969;963–4. 5818337 10.1038/221963a0

[17] Sampaio E, Maris S, Bach-y-Rita P. Brain plasticity: “visual” acuity of blind persons via the tongue. Brain Res. 2001;908:204–7. 11454331 10.1016/s0006-8993(01)02667-1

[18] Abboud S, Hanassy S, Levy-Tzedek S, Maidenbaum S, Amedi A. EyeMusic: Introducing a “visual” colorful experience for the blind using auditory sensory substitution. Restor Neurol Neurosci. 2014;32:247–57. 10.3233/RNN-130338 24398719

[19] Levy-Tzedek S, Hanassy S, Abboud S, Maidenbaum S, Amedi A. Fast, accurate reaching movements with a visual-to-auditory sensory substitution device. Restor Neurol Neurosci. 2012;30:313–23. 10.3233/RNN-2012-110219 22596353

[20] Tyler M, Danilov Y, Bach-Y-Rita P, Medicine R. Closing an Open-Loop Control System: Vestibular Substitution Through the Tongue. J Integr Neurosci. 2003;2:159–64. 15011268 10.1142/s0219635203000263

[21] Auvray M, Hanneton S, O’Regan JK. Learning to perceive with a visuo-auditory substitution system: Localisation and object recognition with “The vOICe.” Perception. 2007;36:416–30. 17455756 10.1068/p5631

[22] Ward J, Meijer P. Visual experiences in the blind induced by an auditory sensory substitution device. Conscious Cogn. 2010;19:492–500. 10.1016/j.concog.2009.10.006 19955003

[23] Bermejo F, Di Paolo E, Hüg MX, Arias C. Sensorimotor strategies for recognizing geometrical shapes: A comparative study with different sensory substitution devices. Frontiers in Psychology. 2015;679. 10.3389/fpsyg.2015.00679 PMC4460306 26106340

[24] Deroy O, Auvray M. Reading the world through the skin and ears: A new perspective on sensory substitution. Front Psychol. 2012;3:1–13. 23162506 10.3389/fpsyg.2012.00457 PMC3491585

[25] Lenay C, Gapenne O, Hanneton S, Marque C, Genouëlle C. Sensory Substitution: Limits and Perspectives. Touching Knowing Cogn Psychol haptic Man Percept. 2003;19:275–92.

[26] Jacobs GH, Williams GA, Cahill H, Nathans J. Emergence of novel color vision in mice engineered to express a human cone photopigment. Science. 2007;315:1723–5. 10.1126/science.1138838 17379811

[27] Norimoto H, Ikegaya Y. Visual cortical prosthesis with a geomagnetic compass restores spatial navigation in blind rats. Current Biology. 2015;1091–5. 10.1016/j.cub.2015.02.063 25843028

[28] Nagel SK, Carl C, Kringe T, Märtin R, König P. Beyond sensory substitution-learning the sixth sense. J Neural Eng. 2005;2:13–26. 10.1088/1741-2560/2/4/R02 16317228

[29] Kärcher SM, Fenzlaff S, Hartmann D, Nagel SK, König P. Sensory Augmentation for the Blind. Front Hum Neurosci. 2012;6:1–15. 22403535 10.3389/fnhum.2012.00037 PMC3290767

[30] Kaspar K, König S, Schwandt J, König P. The experience of new sensorimotor contingencies by sensory augmentation. Conscious Cogn. 2014;28:47–63. 10.1016/j.concog.2014.06.006 25038534 PMC4154453

[31] Schumann F. A Sensorimotor Account of Visual Attention in Natural Behaviour. University of Osnabrück, Germany; 2012. Available from: urn:nbn:de:gbv:700–2013080911054

[32] Ritz T, Dommer DH, Phillips JB. Shedding light on vertebrate magnetoreception. Neuron. 2002;34:503–6. 12062034 10.1016/s0896-6273(02)00707-9

[33] Mora C V., Davison M, Wild JM, Walker MM. Magnetoreception and its trigeminal mediation in the homing pigeon. Nat Neurosci. 2004;432:508–11. 10.1038/nature03077 15565156

[34] Mouritsen H, Ritz T. Magnetoreception and its use in bird navigation. Curr Opin Neurobiol. 2005;15:406–14. 10.1016/j.conb.2005.06.003 16006116

[35] Foulke E. The perceptual basis for mobility. Am Found Blind Res Bull. 1971;23:1–8.

[36] Foulke E. Perception, cognition and the mobility of blind pedestrians Spatial abilities: Development and physiological foundations. San Diego; CA: Academic Press; 1982;55–76.

[37] Loomis JM, Klatzky RL, Golledge RG, Cicinelli JG, Pellegrino JW, Fry PA. Nonvisual navigation by blind and sighted: assessment of path integration ability. J Exp Psychol Gen. 1993;122:73–91. 8440978 10.1037//0096-3445.122.1.73

[38] Walker MP, Stickgold R. Sleep-dependent learning and memory consolidation. Neuron. 2004;44:121–33. 10.1016/j.neuron.2004.08.031 15450165

[39] Maquet P. The role of sleep in learning and memory. Science. 2001;294:1048 10.1126/science.1062856 11691982

[40] Smith C. Sleep states and memory processes in humans: Procedural versus declarative memory systems. Sleep Med Rev. 2001;5:491–506. 10.1053/smrv.2001.0164 12531156

[41] McGaugh JL. Memory-a century of consolidation. Science. 2000;287:248–51. 10634773 10.1126/science.287.5451.248

[42] Smith C. Sleep States and Memory Processes. Behav Brain Res. 1995;69:137–45. 7546305 10.1016/0166-4328(95)00024-n

[43] Fogel SM, Smith CT, Cote KA. Dissociable learning-dependent changes in REM and non-REM sleep in declarative and procedural memory systems. Behav Brain Res. 2007;180:48–61. 10.1016/j.bbr.2007.02.037 17400305

[44] Schwenkreis P, El Tom S, Ragert P, Pleger B, Tegenthoff M, Dinse HR. Assessment of sensorimotor cortical representation asymmetries and motor skills in violin players. Eur J Neurosci. 2007;26:3291–302. 10.1111/j.1460-9568.2007.05894.x 18028115

[45] Maguire E a, Frackowiak RS, Frith CD. Recalling routes around london: activation of the right hippocampus in taxi drivers. J Neurosci. 1997;17:7103–10. 9278544 10.1523/JNEUROSCI.17-18-07103.1997 PMC6573257

[46] Ptito M, Moesgaard SM, Gjedde A, Kupers R. Cross-modal plasticity revealed by electrotactile stimulation of the tongue in the congenitally blind. Brain. 2005;128:606–14. 10.1093/brain/awh380 15634727

[47] Striem-Amit E, Dakwar O, Reich L, Amedi A. The large-Scale Organization of “Visual” Streams Emerges Without Visual Experience. Cereb Cortex. 2012;22:1698–709. 10.1093/cercor/bhr253 21940707

[48] Darwin C. Perception in the Lower Animals. Nature. 1873;7:360–360.

[49] Merkle T, Wehner R. Landmark guidance and vector navigation in outbound desert ants. J Exp Biol. 2008;211:3370–7. 10.1242/jeb.022715 18931310

[50] Mittelstaedt ML, Mittelstaedt H. Homing by path integration in a mammal. Naturwissenschaften. 1980;67:566–7.

[51] v. Frisch K. Die Polarisation des Himmelslichtes als orientierender Faktor bei den Tänzen der Bienen. Experientia. 1949;5:142–8. 18126348 10.1007/BF02174424

[52] Lohmann K, Pentcheff N, Nevitt G, Stetten G, Zimmer-Faust R, Jarrard H, et al Magnetic orientation of spiny lobsters in the ocean: experiments with undersea coil systems. J Exp Biol. 1995;198:2041–8. 9319949 10.1242/jeb.198.10.2041

[53] Wehner R. The ant’s celestial compass system: spectral and polarization channels Orientation and Communication in Anthropods. Basel: Birkhäuser; 1997 p. 145–85.

[54] Cheung A, Vickerstaff R. Finding the way with a noisy brain. PLoS Comput Biol. 2010;6:9–13. 10.1371/journal.pcbi.1000992 PMC2978673 21085678

[55] O’Regan JK. Why red doesn’t sound like a bell: Understanding the feel of consciousness New York, NY, US: Oxford University Press; 2011.

[56] Noë A. Action in perception MIT press; 2004.

[57] Tashakkori A, Teddlie C. Handbook of Mixed Methods in Social & Behavioral Research. Thousand Oaks, CA: Sage; 2010 p. 913

[58] Keyser J. Preparations to study the neural correlates of a vibrotactile sensory augmentation device: Implementation of an fMRI path integration experiment and development of an MRI compatible feelSpace belt. (Bachelor thesis). University of Osnabrueck, Germany; 2010.

[59] Iber C, Ancoli-Israel S, Ph D, Chesson AL, Quan SF. The New Sleep Scoring Manual—The Evidence Behind The Rules. J Clin Sleep Med. 2007;3:107.

[60] Zeitlhofer J, Anderer P, Obergottsberger S, Schimicek P, Lurger S, Marschnigg E, et al Topographic mapping of EEG during sleep. Brain Topogr. 1993;6:123–9. 8123427 10.1007/BF01191077

[61] Werth E, Achermann P, Dijk DJ, Borbély AA. Spindle frequency activity in the sleep EEG: Individual differences and topographic distribution. Electroencephalogr Clin Neurophysiol. 1997;103:535–42. 9402884 10.1016/s0013-4694(97)00070-9

[62] Wolbers T, Wiener JM, Mallot HA, Buchel C. Differential Recruitment of the Hippocampus, Medial Prefrontal Cortex, and the Human Motion Complex during Path Integration in Humans. J Neurosci. 2007;27:9408–16. 10.1523/JNEUROSCI.2146-07.2007 17728454 PMC6673121

[63] Bremmer F, Schlack A, Shah NJ, Zafiris O, Kubischik M, Hoffmann K P, et al Polymodal Motion Processing in Posterior Parietal and Premotor Cortex. Neuron. 2001;29:287–96. 11182099 10.1016/s0896-6273(01)00198-2

[64] Karnath HO, Berger MF, Küker W, Rorden C. The anatomy of spatial neglect based on voxelwise statistical analysis: A study of 140 patients. Cereb Cortex. 2004;14:1164–72. 10.1093/cercor/bhh076 15142954

[65] Dukelow SP, DeSouza JF, Culham JC, van den Berg AV, Menon RS, Vilis T. Distinguishing subregions of the human MT+ complex using visual fields and pursuit eye movements. J Neurophysiol. 2001;86:1991–2000. 11600656 10.1152/jn.2001.86.4.1991

[66] Maldjian JA, Laurienti PJ, Kraft RA, Burdette JH. An automated method for neuroanatomic and cytoarchitectonic atlas-based interrogation of fMRI data sets. Neuroimage. 2003;19:1233–9. 12880848 10.1016/s1053-8119(03)00169-1

[67] Eickhoff SB, Stephan KE, Mohlberg H, Grefkes C, Fink GR, Amunts K, et al A new SPM toolbox for combining probabilistic cytoarchitectonic maps and functional imaging data. Neuroimage. 2005;25:1325–35. 10.1016/j.neuroimage.2004.12.034 15850749

[68] Borkenau P. & Ostendorf F. NEO-Fünf-Faktoren Inventar (NEO-FFI) nach Costa und McCrae. Handanweisung Göttingen: Hogrefe 1993;

[69] Kuhl J. Action versus state orientation: Psychometric properties of the Action Control Scale (ACS-90) Volition and personality: Action versus state orientation. Seattle, Washington D. C.: Hogrefe & Huber; 1994 p. 47–59.

[70] Münzer S, Hölscher C. Entwicklung und Validierung eines Fragebogens zu räumlichen Strategien. Diagnostica. 2011;57:111–25.

[71] Hassenzahl M, Burmester M, Koller F. AttrakDiff: Ein Fragebogen zur Messung wahrgenommener hedonischer und pragmatischer Qualität Mensch & Computer: Interaktion in Bewegung. Stuttgart, Germany: B.G. Teubner; 2003 p. 187–96.

[72] Twisk JWR. Applied multilevel analysis: a practical guide. Technology. 2006. 184 p.

[73] Hennevin E, Leconte P, Bloch V. Effect of acquisition level on the increase of paradoxical sleep duration due to an avoidance conditioning in the rat. CR Hebd Seances Acad Sci, Ser D, Sci Nat. 1971;273:2595–8. 4334535

[74] Leconte P, Hennevin E. Increase of the duration of paradoxical sleep due to learning in the rat. CR Hebd Seances Acad Sci, Ser D, Sci Nat. 1971;273:86–8. 4327135

[75] Campus C, Brayda L, De Carli F, Chellali R, Fama F, Bruzzo C, et al Tactile exploration of virtual objects for blind and sighted people: the role of beta 1 EEG band in sensory substitution and supramodal mental mapping. J Neurophysiol. 2012;107:2713–29. 10.1152/jn.00624.2011 22338024 PMC3362272

[76] Tallon-Baudry C, Bertrand O, Fischer C. Oscillatory synchrony between human extrastriate areas during visual short-term memory maintenance. J Neurosci. 2001;21:RC177 11588207 10.1523/JNEUROSCI.21-20-j0008.2001 PMC6763859

[77] Weiss S, Rappelsberger P. EEG coherence within the 13–18 Hz band as a correlate of a distinct lexical organisation of concrete and abstract nouns in humans. Neurosci Lett. 1996;209:17–20. 8734899 10.1016/0304-3940(96)12581-7

[78] Neuper C, Wörtz M, Pfurtscheller G. ERD/ERS patterns reflecting sensorimotor activation and deactivation. Prog Brain Res. 2006;159:211–22. 10.1016/S0079-6123(06)59014-4 17071233

[79] Perfetti B, Moisello C, Landsness EC, Kvint S, Lanzafame S, Onofrj M, et al Modulation of Gamma and Theta Spectral Amplitude and Phase Synchronization Is Associated with the Development of Visuo-Motor Learning. J Neurosci. 2011;31:14810–9. 10.1523/JNEUROSCI.1319-11.2011 21994398 PMC3206224

[80] Burton H, Videen TO, Raichle ME. Tactile-vibration-activated foci in insular and parietal-opercular cortex studied with positron emission tomography: mapping the second somatosensory area in humans. Somatosens Mot Res. 1993;10:297–308. 8237217 10.3109/08990229309028839

[81] Maldjian JA, Gottschalk A, Patel RS, Pincus D, Detre JA, Alsop DC. Mapping of secondary somatosensory cortex activation induced by vibrational stimulation: An fMRI study. Brain Res. 1999;824:291–5. 10196461 10.1016/s0006-8993(99)01126-9

[82] Johansen-Berg H, Christensen V, Woolrich M, Matthews PM. Attention to touch modulates activity in both primary and secondary somatosensory areas. Neuroreport. 2000;11:1237–41. 10817599 10.1097/00001756-200004270-00019

[83] Dijkerman C, De Haan E. Somatosensory Processes Subserving Perception and Action. Behav Brain Sci. 2007;30:189–239. 10.1017/S0140525X07001392 17705910

[84] Karnath H-O. Spatial neglect-a vestibular disorder? Brain. 2005;129:293–305. 10.1093/brain/awh698 16371409

[85] Wahn B, König P. Audition and vision share spatial attentional resources, yet attentional load does not disrupt audiovisual integration. Front Psychol. 2015;6:1084 10.3389/fpsyg.2015.01084 26284008 PMC4518141

[86] Creem-Regehr SH. Sensory-motor and cognitive functions of the human posterior parietal cortex involved in manual actions. Neurobiol Learn Mem. 2009;91:166–71. 10.1016/j.nlm.2008.10.004 18996216

[87] Iacoboni M. Visuo-motor integration and control in the human posterior parietal cortex: Evidence from TMS and fMRI. Neuropsychologia. 2006;44:2691–9. 10.1016/j.neuropsychologia.2006.04.029 16759673

[88] Culham JC, Valyear KF. Human parietal cortex in action. Curr Opin Neurobiol. 2006;16:205–12. 10.1016/j.conb.2006.03.005 16563735

[89] Corbetta M, Shulman GL, Miezin FM, Petersen SE. Superior parietal cortex activation during spatial attention shifts and visual feature conjunction. Science. 1995;270:802–5. 7481770 10.1126/science.270.5237.802

[90] Wolpert DM, Goodbody SJ, Husain M. Maintaining internal representations: the role of the human superior parietal lobe. Nature Neuroscience. 1998;529–33. 10.1038/2245 10196553

[91] Wander J, Blakely T. Distributed cortical adaptation during learning of a brain-computer interface task. Proc Natl Acad Sci U S A. 2013;110:10818–23. 10.1073/pnas.1221127110 23754426 PMC3696802

[92] Kassubek J, Schmidtke K, Kimmig H, Lücking CH, Greenlee MW. Changes in cortical activation during mirror reading before and after training: An fMRI study of procedural learning. Cogn Brain Res. 2001;10:207–17. 10.1016/s0926-6410(00)00037-9 11167046

[93] Kelly AMC, Garavan H. Human functional neuroimaging of brain changes associated with practice. Cereb Cortex. 2005;15:1089–102. 10.1093/cercor/bhi005 15616134

[94] Grill-Spector K, Henson R, Martin A. Repetition and the brain: Neural models of stimulus-specific effects. Trends Cogn Sci. 2006;10:14–23. 10.1016/j.tics.2005.11.006 16321563

[95] Peterson S.E., van Mier H., Fiez JA., Raichle, M. SE. The effects of practice on the functional anatomy of task performance. Proc Natl Acad Sci U S A. 1998;95:853–60. 9448251 10.1073/pnas.95.3.853 PMC33808

[96] Poldrack RA. Imaging Brain Plasticity: Conceptual and Methodological Issues—A Theoretical Review. Neuroimage. 2000;12:1–13. 10.1006/nimg.2000.0596 10875897

[97] Jonides J. How does practice makes perfect? Nat Neurosci. 2004;7:10–1. 10.1038/nn0104-10 14699412

[98] Clark D, Schumann F, Mostofsky SH. Mindful movement and skilled attention. Front Hum Neurosci. 2015;9:1–23. 26190986 10.3389/fnhum.2015.00297 PMC4484342

[99] Bremmer F, Duhamel JR, Ben Hamed S, Graf W. Heading encoding in the macaque ventral intraparietal area (VIP). Eur J Neurosci. 2002;16:1554–68. 12405970 10.1046/j.1460-9568.2002.02207.x

[100] Orban G a, Dupont P, De Bruyn B, Vogels R, Vandenberghe R, Mortelmans L. A motion area in human visual cortex. Proc Natl Acad Sci U S A. 1995;92:993–7. 7862680 10.1073/pnas.92.4.993 PMC42623

[101] Tanji J. The supplementary motor area in the cerebral cortex. Neurosci Res. 1994;19:251–68. 8058203 10.1016/0168-0102(94)90038-8

[102] Penfield W. The Supplementary Motor Area of the Cerebral Cortex. AMA Arch Neurol Psychiatry. American Medical Association; 1951;66:289 14867993 10.1001/archneurpsyc.1951.02320090038004

[103] Cunnington R, Windischberger C, Moser E. Premovement activity of the pre-supplementary motor area and the readiness for action: Studies of time-resolved event-related functional MRI. Hum Mov Sci. 2005;24:644–56. 10.1016/j.humov.2005.10.001 16337295

[104] Rizzolatti G, Fadiga L, Gallese V, Fogassi L. Premotor cortex and the recognition of motor actions. Cogn Brain Res. 1996;3:131–41. 10.1016/0926-6410(95)00038-0 8713554

[105] Blakemore SJ, Frith CD, Wolpert DM. The cerebellum is involved in predicting the sensory consequences of action. Neuroreport. 2001;12:1879–84. 11435916 10.1097/00001756-200107030-00023

[106] Iglói K, Doeller CF, Paradis A-L, Benchenane K, Berthoz A, Burgess N, et al Interaction Between Hippocampus and Cerebellum Crus I in Sequence-Based but not Place-Based Navigation. Cereb Cortex. 2014;132. 10.1093/cercor/bhu132 PMC4886832 24947462

[107] Iaria G, Petrides M, Dagher A, Pike B, Bohbot VD. Cognitive strategies dependent on the hippocampus and caudate nucleus in human navigation: variability and change with practice. J Neurosci. 2003;23:5945–52. 12843299 10.1523/JNEUROSCI.23-13-05945.2003 PMC6741255

[108] Hartley T, Maguire EA, Spiers HJ, Burgess N. The well-worn route and the path less traveled: distinct neural bases of route following and wayfinding in humans. Neuron. 2003;37:877–88. 12628177 10.1016/s0896-6273(03)00095-3

[109] Voss P, Fortin M, Corbo V, Pruessner JC, Lepore F. Assessment of the caudate nucleus and its relation to route learning in both congenital and late blind individuals. BMC Neuroscience. 2013;14:113 10.1186/1471-2202-14-113 24093549 PMC3851784

[110] Spiers HJ, Maguire E a. Thoughts, behaviour, and brain dynamics during navigation in the real world. Neuroimage. 2006;31:1826–40. 10.1016/j.neuroimage.2006.01.037 16584892

[111] Iaria G, Chen J-K, Guariglia C, Ptito A, Petrides M. Retrosplenial and hippocampal brain regions in human navigation: complementary functional contributions to the formation and use of cognitive maps. Eur J Neurosci. 2007;25:890–9. 10.1111/j.1460-9568.2007.05371.x 17298595

[112] Wolbers T. Dissociable Retrosplenial and Hippocampal Contributions to Successful Formation of Survey Representations. J Neurosci. 2005;25:3333–40. 10.1523/JNEUROSCI.4705-04.2005 15800188 PMC6724902

[113] Maguire EA. Knowing Where and Getting There: A Human Navigation Network. Science. 1998;280:921–4. 9572740 10.1126/science.280.5365.921

[114] Morgan LK, MacEvoy SP, Aguirre GK, Epstein RA. Distances between Real-World Locations Are Represented in the Human Hippocampus. J Neurosci. 2011;31:1238–45. 10.1523/JNEUROSCI.4667-10.2011 21273408 PMC3074276

[115] Fujita N, Klatzky RL, Loomis JM, Golledge RG. The Encoding-Error Model of Pathway Completion without Vision. Geogr Anal. 1993;25:295–314.

[116] Wiener J., Mallot H. Path Complexity Does Not Impair Visual Path Integration. Spat Cogn Comput. 2006;6:295–308.

[117] Wahn B, König P. Vision and Haptics Share Spatial Attentional Resources and Visuotactile Integration Is Not Affected by High Attentional Load. Multisens Res. 2015;28:371–92. 26288905 10.1163/22134808-00002482

[118] Wahn B, König P. Attentional resource allocation in visuotactile processing depends on the task, but optimal visuotactile integration does not depend on attentional resources. Front Integr Neurosci. 2016;10:13 10.3389/fnint.2016.00013 27013994 PMC4781873

[119] White BW, Saunders F a., Scadden L, Bach-Y-Rita P, Collins CC. Seeing with the skin. Percept Psychophys. 1970;7:23–7.

[120] Lenay C, Canu S, Villon P. Technology and perception: the contribution of sensory substitution\nsystems. Proc Second Int Conf Cogn Technol Humaniz Inf Age. 1997;44–53.

[121] Ehinger B V, Fischer P, Gert AL, Kaufhold L, Weber F, Pipa G, et al Kinesthetic and vestibular information modulate alpha activity during spatial navigation: a mobile EEG study. Front Hum Neurosci. 2014;8:71 10.3389/fnhum.2014.00071 24616681 PMC3934489

[122] Amedi A, Malach R, Hendler T, Peled S, Zohary E. Visuo-haptic object-related activation in the ventral visual pathway. Nat Neurosci. 2001;4:324–30. 10.1038/85201 11224551

[123] Amedi A, Jacobson G, Hendler T, Malach R, Zohary E. Convergence of visual and tactile shape processing in the human lateral occipital complex. Cereb Cortex. 2002;12:1202–12. 12379608 10.1093/cercor/12.11.1202

[124] Burton H, Snyder AZ, Diamond JB, Raichle ME. Adaptive Changes in Early and Late Blind: A fMRI Study of Verb Generation to Heard Nouns Methods. 2005;3359–71. 10.1152/jn.00129.2002 PMC3704164 12466452

[125] Röder B, Stock O, Bien S, Neville H, Rösler F. Speech processing activates visual cortex in congenitally blind humans. Eur J Neurosci. 2002;16:930–6. 12372029 10.1046/j.1460-9568.2002.02147.x

[126] Sadato N, Pascual-Leone A, Grafman J, Ibanez V, Deiber MP, Dold G, et al Activation of the primary visual cortex by Braille reading in blind subjects. Nature. 1996;380:526–8. 10.1038/380526a0 8606771

[127] Cohen LG, Celnik P, Pascual-Leone A, Corwell B, Falz L, Dambrosia J, et al Functional relevance of cross-modal plasticity in blind humans. Nature. 1997;389:180–3. 10.1038/38278 9296495

[128] Dayan E, Cohen LG. Neuroplasticity subserving motor skill learning. Neuron. 2011;72:443–54. 10.1016/j.neuron.2011.10.008 22078504 PMC3217208

[129] Wiesel TN, Hubel DH. Effects if visual deprivation on morphology and physiology of cells in the cat’s lateral geniculate body. J Neurophysiol. 1963;26:978–93. 14084170 10.1152/jn.1963.26.6.978

[130] Büchel C, Price C, Frackowiak RSJ, Friston K. Different activation patterns in the visual cortex of late and congenitally blind subjects. Brain. 1998;121:409–19. 9549517 10.1093/brain/121.3.409

[131] Sadato N, Okada T, Honda M, Yonekura Y. Critical period for cross-modal plasticity in blind humans: a functional MRI study. Neuroimage. 2002;16:389–400. 10.1006/nimg.2002.1111 12030824

[132] Röder B, Rösler F, Spence C. Early Vision Impairs Tactile Perception in the Blind. Curr Biol. 2004;14:121–4. 14738733

[133] Tse ZTH, Janssen H, Hamed A, Ristic M, Young I, Lamperth M. Magnetic resonance elastography hardware design: a survey. Proc Inst Mech Eng H. 2009;223:497–514. 19499839 10.1243/09544119JEIM529

[134] Gassert R, Yamamoto A, Chapuis D, Dovat L, Bleuler H, Burdet E. Actuation Methods for Applications in MR Environments. Concepts Magn Reson Part B Magn Reson Eng. 2006;29:191–209.

[135] Francis ST, Kelly EF, Bowtell R, Dunseath WJ, Folger SE, McGlone F. fMRI of the responses to vibratory stimulation of digit tips. Neuroimage. 2000;11:188–202. 10.1006/nimg.2000.0541 10694461

[136] Angelaki DE, Cullen KE. Vestibular System: The Many Facets of a Multimodal Sense. Annu Rev Neurosci. 2008;31:125–50. 10.1146/annurev.neuro.31.060407.125555 18338968

[137] Jones LA, Sarter NB. Tactile Displays: Guidance for Their Design and Application. Hum Factors. 2008;50:90–111. 18354974 10.1518/001872008X250638

[138] Cholewiak RW, Brill JC, Schwab A. Vibrotactile localization on the abdomen: effects of place and space. Percept Psychophys. 2004;66:970–87. 15675645 10.3758/bf03194989

[139] Agnew HW, Webb WB, Williams RL. The first night effect: an EEG study of sleep. Psychophysiology. 1966;2:263–6. 5903579 10.1111/j.1469-8986.1966.tb02650.x

[140] Ellmore T, McNaughton B. Human Path Integration by Optic Flow. Spat Cogn Comput. 2004;4:255–72.

